# HIV Impacts CD34^+^ Progenitors Involved in T-Cell Differentiation During Coculture With Mouse Stromal OP9-DL1 Cells

**DOI:** 10.3389/fimmu.2019.00081

**Published:** 2019-01-29

**Authors:** Tetsuo Tsukamoto

**Affiliations:** ^1^The Kirby Institute for Infection and Immunity in Society, University of New South Wales, Sydney, NSW, Australia; ^2^Center for AIDS Research, Kumamoto University, Kumamoto, Japan; ^3^Department of Immunology, Faculty of Medicine, Kindai University, Osaka, Japan

**Keywords:** human immunodeficiency virus (HIV), acquired immunodeficiency syndrome (AIDS), hematopoietic stem/progenitor cells (HSPCs), lymphopoiesis, CXCR4

## Abstract

HIV-1 causes the loss of CD4^+^ T cells via depletion or impairment of their production. The latter involves infection of thymocytes, but the involvement of hematopoietic CD34^+^ cells remains unclear even though HIV-positive patients frequently manifest myelosuppression. In order to have a closer look at the impact of HIV-1 on T-lineage differentiation, this study utilized the OP9-DL1 coculture system, which supports *in vitro* T-lineage differentiation of human hematopoietic stem/progenitor cells. In the newly developed *in vitro* OP9-DL1/HIV-1 model, cord-derived CD34^+^ cells were infected with CXCR4-tropic HIV-1_NL4−3_ and cocultured. The HIV-infected cocultures exhibited reduced CD4^+^ T-cell growth at weeks 3–5 post infection compared to autologous uninfected cocultures. Further assays and analyses revealed that CD34^+^CD7^+^CXCR4^+^ cells can be quickly depleted as early as 1 week after infection of the subset, and this was accompanied by the emergence of rare CD34^+^CD7^+^CD4^+^ cells. A subsequent theoretical model analysis suggested potential influence of HIV-1 on the differentiation rate or death rate of lymphoid progenitor cells. These results indicate that CXCR4-tropic HIV-1 strains may impact the dynamics of CD34^+^CD7^+^ lymphoid progenitor cell pools, presumably leading to impaired T-cell production potential.

## Introduction

Human immunodeficiency virus (HIV) infection is associated with hematological changes (1). Antiretroviral therapy is effective in controlling viremia and treating acquired immunodeficiency syndrome (AIDS). However, some patients do not experience sufficient T-cell immune restoration despite being aviremic during treatment (2). Regarding T-cell generation in the thymus, HIV-infected patients may display decreased thymopoiesis (3), and thymic dysfunction during HIV infection may be associated with rapid progression in infants with prenatal HIV infection (4). A previous report tested coculture of CD34^+^ and fetal thymic epithelial cells in the presence or absence of HIV-1, observing that infection led to the inhibition of thymocyte maturation at early stages (CD44^+^CD25^−^CD3^−^) (5). On the other hand, bone marrow abnormalities in HIV-infected individuals may result in not only reduced T-cell production but abnormalities in erythropoiesis, myelopoiesis, and megakaryopoiesis (6, 7). A previous study revealed depletion of CD34^+^CD4^+^ cells in bone marrow from HIV-infected patients. However, the study failed to provide evidence for HIV infection of these cells (8). A number of evidences have recently been presented on hematopoietic progenitors harboring CXCR4-tropic and CCR5-tropic HIV genomes as potentially long-lasting viral reservoirs (9).

Despite that HIV-1 may infect human CD34^+^ hematopoietic stem/progenitor cells (HSPCs) *in vitro* (10, 11), HSPCs have multiple mechanisms to limit HIV infection. One mechanism of limitation is the low expression levels of CD4, CXCR4, and CCR5 on CD34^+^CD133^+^ stem/progenitor cells, although these cells express CXCR4 more widely than CCR5 (11). In addition, a recent report has indicated mechanisms that restrict HIV-1 prior to integration of viral DNA in cord-derived CD34^+^ cells (12). These various mechanisms of HIV infection limitation have prevented researchers from detailed *in vitro* analysis of CD34^+^ cells in the presence of HIV-1. To overcome these limitations, a novel method to mediate HIV-1 entry to CD34^+^ cells using RetroNectin (RN), a recombinant fibronectin fragment that enhances retroviral-mediated gene transduction by aiding the co-localization of target cells and virions, was described (13). This method enables long-term coculture of HIV-infected HSPCs with the OP9-DL1 cells.

The OP9-DL1 and OP9-DL4 cell lines are widely used to mimic thymopoiesis *in vitro*. They were derived from the OP9 mouse stromal cell line via transduction with a Notch ligand called delta-like 1 (DL1) or DL4 (14, 15). The ability of OP9-DL1 cells to support thymopoiesis was first demonstrated in coculture with mouse cells (14). The cell line is also known to support the differentiation of human CD34^+^ HSPCs into thymocytes and T cells (16). There is evidence that OP9-DL1 cells produce stromal derived factor-1 (SDF-1, also known as CXCL12), a ligand for CXCR4 (17, 18). Therefore, by producing SDF-1, OP9-DL1 cells not only could favor and support the differentiation of human CD34^+^ HSPCs into thymocytes and T cells, but also could trigger proviral DNA expression, then promoting HIV-1 replication in the model used in this work (19). Although OP9-DL4 cells can induce differentiation into both specific myeloid cells and T-lineage cells, OP9-DL1 cells only permit the differentiation of T-lineage cells while inhibiting B cells and myeloid cells (20). Thus, the OP9-DL1 coculture system is used to investigate events associated with T-lineage differentiation. OP9-DL1 is also able to support the generation of CD34^+^CD7^+^ lymphoid progenitors that can engraft the thymus of immunodeficient mice (21, 22).

T-lineage lymphopoiesis starts in the bone marrow. A subset of bone marrow cells called common lymphoid progenitors (CLPs), characterized by the surface expression of CD34, CD7, and CD10, are generated from multipotent progenitors and are considered to be the most immature lymphoid-committed progenitors (23–25). CD34^+^CD7^+^ cells are reported to be about 2.7% of bone marrow CD34^+^ cells (26). CLPs migrate to thymus and differentiate to early thymic progenitors called Pro-T cells expressing CD34, CD45RA, and CD7 (21). Following this, all the important events in T-cell development (double-negative thymocytes, double positive thymocytes, and CD4^+^ or CD8^+^ naïve T cells) occur in the thymus (27). CD7 is expressed on the surface of lymphoid progenitors, T, NK, and pro-B cells and important for lymphoid development (28). CD7 is highly expressed on naïve and memory T cells (29, 30), while CD7^low/−^ T subsets may contain effector T cells (30). CD8^+^ T cells from HIV-infected patients show down modulation of CD7 with expansion of CD7^low/−^ subsets (31). CD7^−^ T-cells also increase with aging (32).

The chemokine receptors CXCR4 and CCR5 are common coreceptors for HIV-1 (33). Control of CCR5-tropic strains of HIV-1 is usually considered a better correlate of good clinical outcomes than the control of CXCR4-tropic HIV-1 strains (34). This is because memory CD4^+^ T cells express higher levels of CCR5, thus making them susceptible to CCR5-tropic HIV infection and subsequent depletion (35). Consequently, CCR5 may appear to be more closely involved in the immunopathogenesis of HIV infection than CXCR4 (36). However, some data indicating similar cytotoxicity in CD4^+^ T cells caused by CXCR4- and CCR5-tropic HIV-1 strains has been reported. Hence, both CCR5- and CXCR4-tropic HIV-1 strains can cause CD4^+^ T cell depletion following infection via mucosal routes in a humanized mouse model (37). Furthermore, CXCR4-tropic viral strains are associated with a more rapid progression of the HIV-1 disease. Hence, CXCR4-tropic isolates predominate in most infected individuals (38, 39), and the conversion of HIV-1 envelope (Env) tropism from CCR5 to CXCR4 is related to rapid disease progression due to a more reduced CD4^+^ T cell counts with subsequent poor clinical prognosis (40–49). Indeed, rapid decline of CD4 counts and disease progression has also been reported when infection is initiated by dual CCR5/CXCR4 dual-tropic or CXCR4-tropic HIV strains (44, 50). On the other hand, the emergence of CXCR4-tropic HIV-1 may be associated with poor prognosis (51, 52). Therefore, this poor prognosis is crucial for the progression of the infection and pathogenesis *in vivo*, since X4-tropic HIV-1 viral strains, through infecting multipotent hematopoietic stem and progenitor cells, are directly involved in the maintenance of the long-lived cellular reservoir of latently integrated HIV-1 (11). The biological functions of CXCR4 have been well documented in developmental biology and hematology. CXCR4 interacts with SDF-1 and allows CXCR4-expressing cells to home in on loci in which SDF-1 is highly expressed (17, 53, 54). SDF-1 and CXCR4 are essential in human stem cell homing and repopulation of the host with differentiated hematopoietic cells (55, 56). SDF-1 is also produced by thymic epithelial cells, and it plays an important role in migration of immature progenitors in the thymus (57).

The above evidences implicate the potential influence of CXCR4-tropic HIV-1 infection on hematopoiesis and T-cell development (58). A prior study sought to clarify the effect of CXCR4-tropic simian-human immunodeficiency virus infection on T-lineage cell production in the thymi of newborn rhesus macaques (59). However, it is not realistic to closely follow *in vivo* bone marrow/thymus events in HIV-infected individuals. Instead, humanized mouse models can be beneficial for this purpose (60, 61). Moreover, an easy-to-use *ex-vivo* model may be helpful for closely monitoring the differentiation of HSPCs into T-lineage cells in the presence of HIV-1. Although previous *in vitro* assays demonstrated susceptibility of HSPCs to HIV-1 infection and suggested pathogenic roles of CXCR4-tropic HIV-1, some of those assays relied on strong *in vitro* cytokine stimulation of HSPCs that may cause significant upregulation of HIV-1 (co)receptors (10, 11). The present study aimed to develop a novel *in vitro* model to follow up T-lineage differentiation more closely by using the OP9-DL1 coculture system, and determine the *in vitro* fate of CD34^+^ progenitor cells and derivatives exposed to HIV-1.

## Materials and Methods

### Virus Stocks

Stocks of HIV-1_NL4−3_ were produced via lipid-based transfection of 293T cells with the molecular clone DNA pNL4-3 (62) using the HilyMax reagent (Dojindo Laboratories, Kumamoto, Japan). After transfection, the culture supernatant was collected, aliquoted (500 μL/ tube) in screw capped 1.5 mL tubes and stored in a −80°C freezer in a biosafety level 3 (BSL-3) laboratory located at Center for AIDS Research, Kumamoto University. All manipulations using the virus stocks were performed in the BSL-3 lab. Viral loads ranged roughly from 700 to 800 ng/mL as determined by an HIV p24 enzyme-linked immunosorbent assay (ELISA) kit (ZeptoMetrix, NY, USA).

### Cells

Umbilical cord blood samples were collected at Fukuda Hospital, Kumamoto, Japan after obtaining informed consent. Cord blood mononuclear cells were isolated using Pancoll (PAN-Biotech GmbH, Aidenbach, Germany) and centrifugation at 800 × g for 20 min. Cells were resuspended in phosphate-buffered saline (PBS) supplemented with 0.2 % bovine serum albumin (BSA) and 2 mM EDTA, labeled with human CD34 microbeads (Miltenyi Biotec, NSW, Australia) for 15 min and washed, and isolated using LS columns (Miltenyi Biotec) according to the manufacturer's protocol. The purity of CD34^+^ cells consistently exceeded 92% by flow cytometry. For purifying CD34^−^ cells, the CD34^−^ fraction obtained by the LS column sorting was further depleted of residual CD34^+^ cells by using LD columns (Miltenyi Biotec). The OP9-DL1 cell line was provided for this study by the Center for AIDS Research, Kumamoto University, Japan, which had been generated via stable retroviral transduction of the OP9 cell line (RCB1124, Riken, Tsukuba, Japan) with human DL1 as previously described (63). OP9-DL1 cells serve as the provider of both DL1 and SDF-1 signals (18). The cell line was tested and confirmed for its support for the differentiation of human CD34^+^ cells to thymocytes and T cells (Figures 2, 3) but not to B cells or myeloid cells (data not shown). The cell line was maintained in α-MEM medium (Wako Pure Chemical Industries, Osaka, Japan) supplemented with 10% heat inactivated fetal bovine serum (FBS, GE Healthcare, Tokyo, Japan). This was called OP9-DL1 culture medium.

### Antibodies

The antibody concentrations are indicated in ng or test (as indicated by the manufacturers if the real concentration for the product is not available) as follows. Anti-human CD8 Brilliant Violet (BV) 510 (clone RPA-T8, used at 0.1 test/50 μL), anti-human CD3 PE-Cy7 (clone UCHT1, used at 0.1 test/50 μL) and anti-human CD34 APC (clone 8G12, used 50 ng/50 μL) were purchased from BD Biosciences (NSW, Australia). Anti-human CD4 PE-Cy7 (clone OKT4, used at 150 ng/50 μL), anti-human CD4 PerCP-Cy5.5 (clone OKT4, used at 25 ng/50 μL) and anti-human CXCR4 BV421 (clone 12G5, 50 ng/50 μL) were purchased from BioLegend (CA, USA). Anti-human CD3 ECD (clone UCHT1, used at 0.05 test/50 μL) and anti-HIV-1 p24 PE (clone FH190-1-1, also known as KC57 RD1, used at 0.1 test/50 μL) were purchased from Beckman Coulter (NSW, Australia). Anti-human CD7 FITC (clone CD7-6B7, used at 50 ng/50 μL) was purchased from CALTAG Laboratories (CA, USA). Optimal concentrations per test were determined by flow cytometry prior to the experiments.

### HIV Infection

The method for *in vitro* HIV infection of CD34^+^ cells was described previously (13). To infect primary cord-derived CD34^+^ cells with HIV-1_NL4−3_, a 48-well plate (Corning) was treated overnight with RetroNectin (Takara Bio, Kusatsu, Japan) at a concentration of 10 μg/mL. CD34^+^ cells were re-suspended in the OP9-DL1 medium, seeded at 2 × 10^5^ per well in the coated plate and infected with 200 ng (p24) of HIV-1_NL4−3_ using spinoculation at 1,200 × *g* at 34°C for 30 min. Cells were further cultured overnight and cocultured with OP9-DL1 cells as described below. For HIV infection of OP9-cocultured human cells, the cell concentration was adjusted to 5 × 10^5^ per well.

### Coculture of Human Cells With OP9-DL1

The OP9-DL1 coculture experiment was performed following a previously published protocol with modifications (63). Briefly, 2 × 10^5^ HIV-infected or uninfected cord-derived CD34^+^ cells were seeded in a 6-well plate (Corning, VIC, Australia) containing a monolayer of OP9-DL1 cells that had been seeded at 1 × 10^5^ cells per well a day before passage. The coculture was maintained for 5 weeks in α-MEM medium supplemented with 20% heat inactivated FBS, 5 ng/mL recombinant human FMS-like tyrosine kinase 3 ligand (Flt-3L) (R&D Systems, MN, USA) and 5 ng/mL recombinant human interleukin (IL)-7 (Miltenyi Biotec). This was called coculture medium. Cells were passaged weekly with vigorous pipetting and filtering through a 70-μm membrane and cocultured again with a fresh monolayer of OP9-DL1 cells. A portion (20%) of the collected cells was analyzed by flow cytometry. The medium was washed out at each passage by centrifugation at 400 × g for 5 min, and changed to the fresh coculture medium.

### Flow Cytometry

Cells were collected and washed by phosphate-buffered saline (PBS) containing 0.2% bovine serum albumin (staining buffer). Cells were then resuspended in 50 μl of the staining buffer containing the surface-staining antibodies against CD34, CXCR4, CD7, CD3, CD4, or CD8 at the indicated concentrations. After incubation at room temperature for 20 minutes, cells were washed by adding 100 μl of the staining buffer followed by centrifugation at 1,000 × g for 2 min. After discarding the supernatants, cells were fixed/permealized at room temperature for 20 min, and intracellularly stained with anti-HIV p24 PE at room temperature for 20 min by using Cytofix/Cytoperm reagents (BD Biosciences) following the manufacturer's instructions and previously established procedures (64). Surface and intracellular antigen expression was analyzed using a FACS LSR II (BD Biosciences), FACS Diva v6.0 software (BD Biosciences) and FlowJo v10.4 software (FlowJo, OR, USA). Dead cells were discriminated using Live/Dead Fixable Near-IR Dead Cell Stains (Thermo Fisher Scientific, VIC, Australia). Live cells were further gated to exclude doublets from the analysis by plotting FSC-H and FSC-A.

### Quantitation of HIV-1 p24 in Co-culture Supernatants

Prior to passage, co-culture supernatants were collected every week from 1 to 5 weeks after infection, plated in 96 U-bottom plates, and stored in −80°C until analysis. The supernatant was used undiluted or diluted by the OP9-DL1 medium, and HIV-1 p24 content was measured by using ELISA.

### PCR Analysis of HIV DNA

Cellular DNA was extracted using a Kaneka Easy DNA Extraction Kit (Kaneka, Takasago, Japan). DNA extraction was followed by the non-quantitative PCR analysis using an HIV *gag* primer set (sense: 5′-AGTGGGGGGACATCAAGCAGCCATGCAAAT-3′, antisense: 5′-TACTAGTAGTTCCTGCTATGTCACTTCC-3′) as described previously (65). The oligo DNAs were purchased from Sigma-Aldrich (Tokyo, Japan).

### Statistical Analysis

Statistical analysis was performed using GraphPad Prism software version 7.0 (GraphPad Software, CA, USA). Statistical significance was defined as *P* < 0.05. Comparisons between HIV-infected and uninfected samples were performed using Wilcoxon's matched-pairs signed rank test, unless otherwise stated. Multiple comparison analyses were done, if necessary, using Dunn's method. Spearman's correlation coefficients were calculated for correlation analyses.

### Theoretical Modeling

A theoretical model was constructed to fit the results of this study. As there were few previously published data to help determine the parameters for such model, except for some estimates on the self-renewal rates of hematopoietic stem cells *in vivo* estimated as approximately 2.5 weeks in mice, 8.3–10 weeks in cats, and 25–50 weeks in humans (66, 67), the parameters were set to fit the current data on *in vitro* differentiation of CD34^+^ cells in the presence or absence of HIV-1. There was an assumption that CXCR4-expressing cells may compete with each other for the CXCR4/SDF-1 axis-dependent differentiation to T-lineage cells. Values were calculated from the set of ordinary differential equations described in Figure S6 by using GNU Octave version 4.2.1, and visualized by gnuplot version 5.2.

### Data Availability

The data that support the findings of this study are available from the corresponding author upon request.

## Results

### Persistent HIV-1 Infection Observed in OP9-DL1 Cocultures With Cord-Derived CD34^+^ Cells

**Figure 1:**
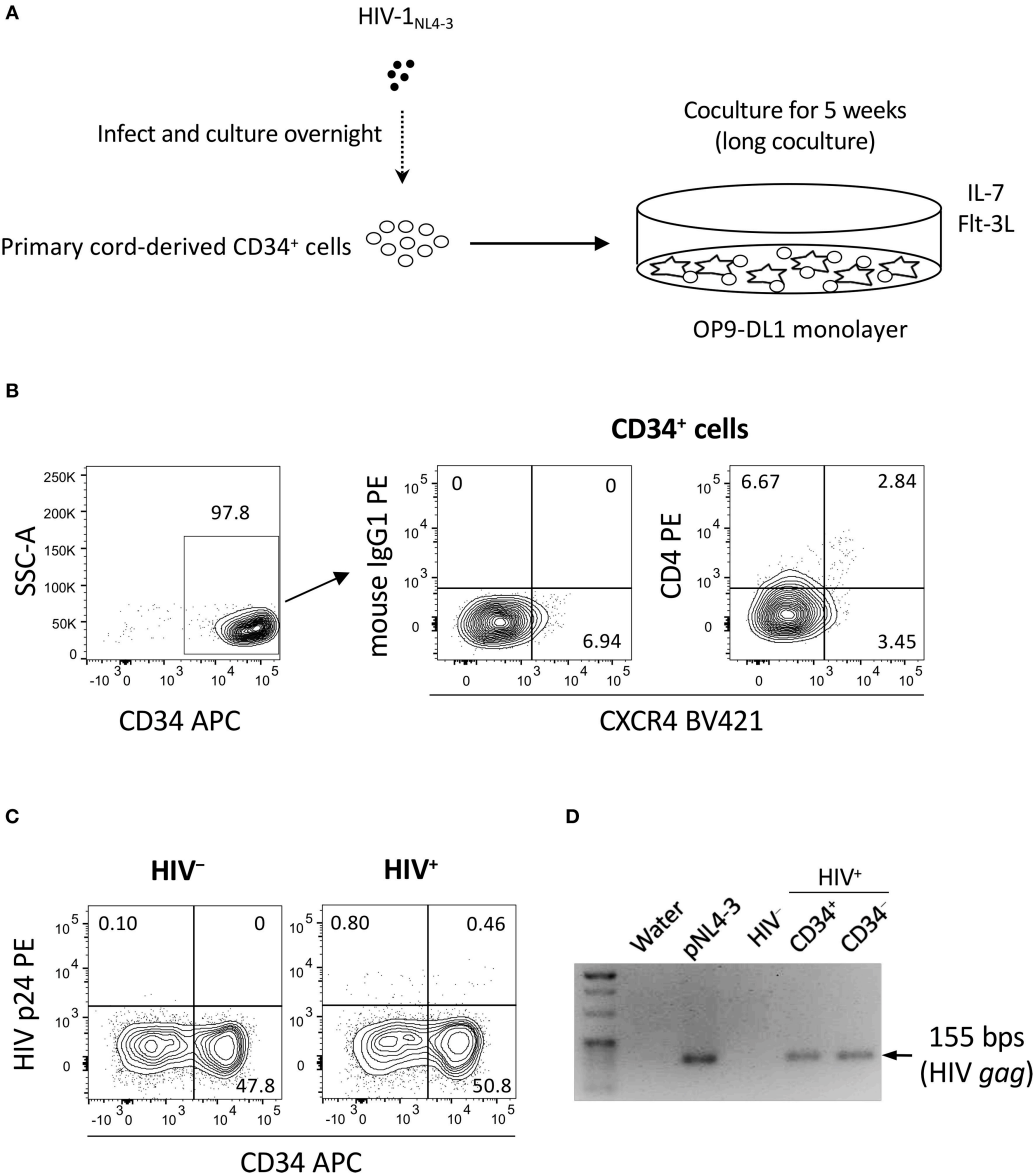
The design of experiment 1 (long coculture). Primary umbilical cord-derived CD34^+^ cells were susceptible to HIV infection, and coculture of HIV-infected primary cord-derived CD34^+^ cells with OP9-DL1 cells resulted in persistent viral replication for 5 weeks (*n* = 12). **(A)** Schematic representation of experiment 1. Cord-derived CD34^+^ cells were infected with HIV-1_NL4−3_ and cocultured with OP9-DL1 cells for 5 weeks. **(B)** Primary cord-derived CD34^+^ cells were gated and tested for CD4 and CXCR4 expression by flow cytometry. Representative plots are shown. The CD4 signal levels were compared to the signal levels of a PE-conjugated mouse isotype control antibody. **(C)** Intracellular HIV p24 expression was tested 1 week after infection. HIV p24^+^ cells were found in both the CD34^+^ and CD34^−^ fractions of HIV-infected samples. Representative plots are shown. **(D)** The CD34^+^ and CD34^−^ fractions of an HIV-infected sample were separated using the CD34 microbead method. Total DNA was isolated and analyzed by PCR. HIV gag DNA was detected in both the CD34^+^ and CD34^−^ fractions. A PCR sample using the HIV molecular plasmid pNL4-3 was used as a positive control, while a PCR sample using an OP9-DL1 cocultured cells without HIV infection was used as a negative control. Statistical analyses were performed using the Wilcoxon matched-pairs signed rank test.

**Figure 2:**
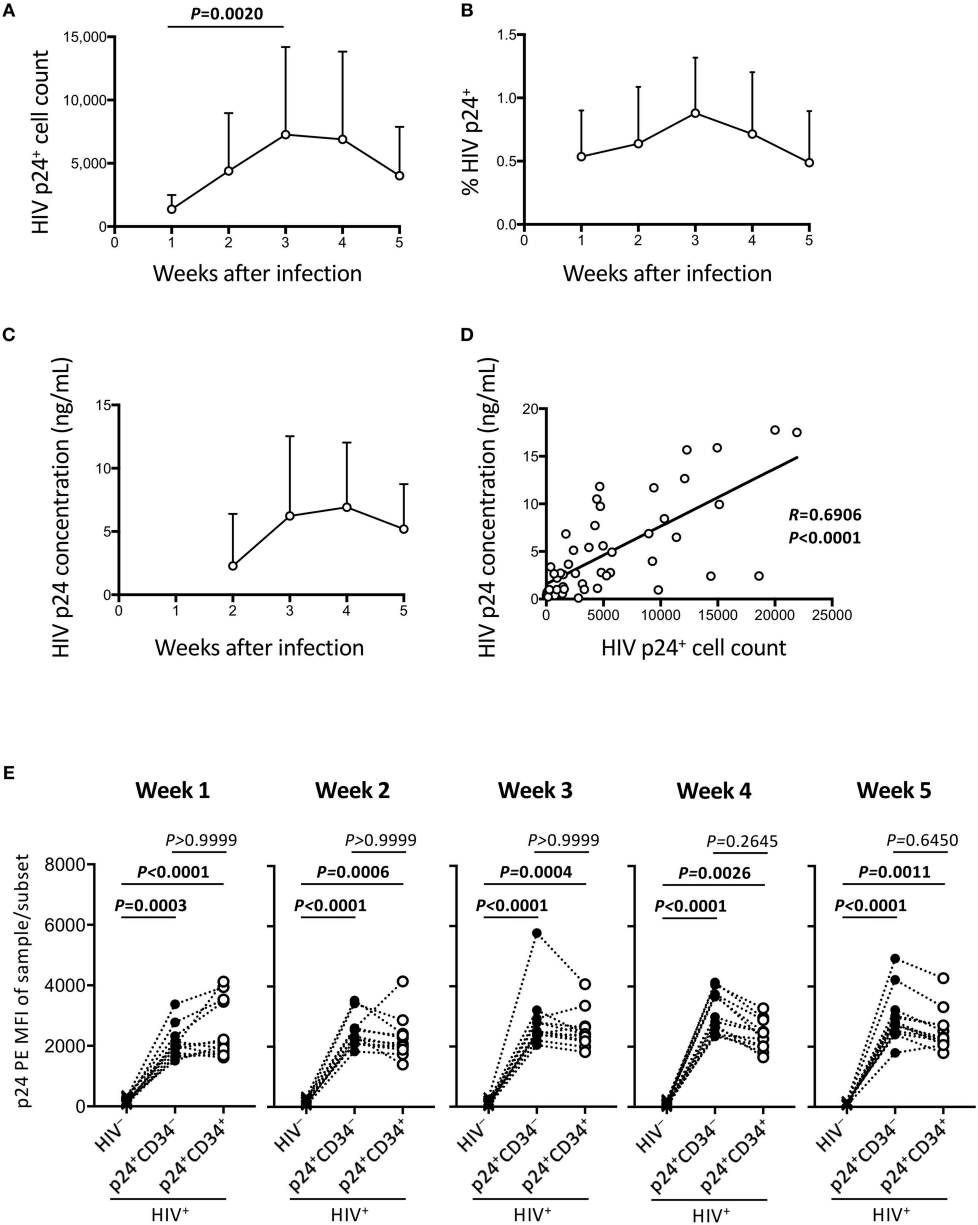
Persistent HIV replication in the OP9-DL1 cocultures of experiment 1 (long coculture). HIV infection of cord-derived CD34^+^ cells and derivatives were evaluated (*n* = 12 unless otherwise indicated). **(A)** HIV p24^+^ cell counts at weeks 1–5 post-infection. **(B)** Percentage of HIV p24^+^ cells in HIV^+^ OP9-DL1 coculture samples tested weekly by intracellular staining and flow cytometry. **(C)** Coculture supernatants of HIV-infected samples were tested for HIV p24 concentrations by ELISA at weeks 2–5. **(D)** Correlation analysis between the frequencies of HIV p24^+^ cells and HIV p24 concentrations. The weeks 2–5 data in **(A,C)** are included (*n* = 48). Spearman's correlation coefficients are shown. Comparisons were performed using the Wilcoxon matched-pairs signed rank test. **(E)** p24 PE mean fluorescence intensities (MFIs) of p24^+^CD34^−^ and p24^+^CD34^+^ cells in HIV-infected samples were compared to p24-PE MFIs of autologous uninfected samples at weeks 1–5 post infection. Statistical analysis was performed using the multiple comparison test with Dunn's method.

**Figure 3:**
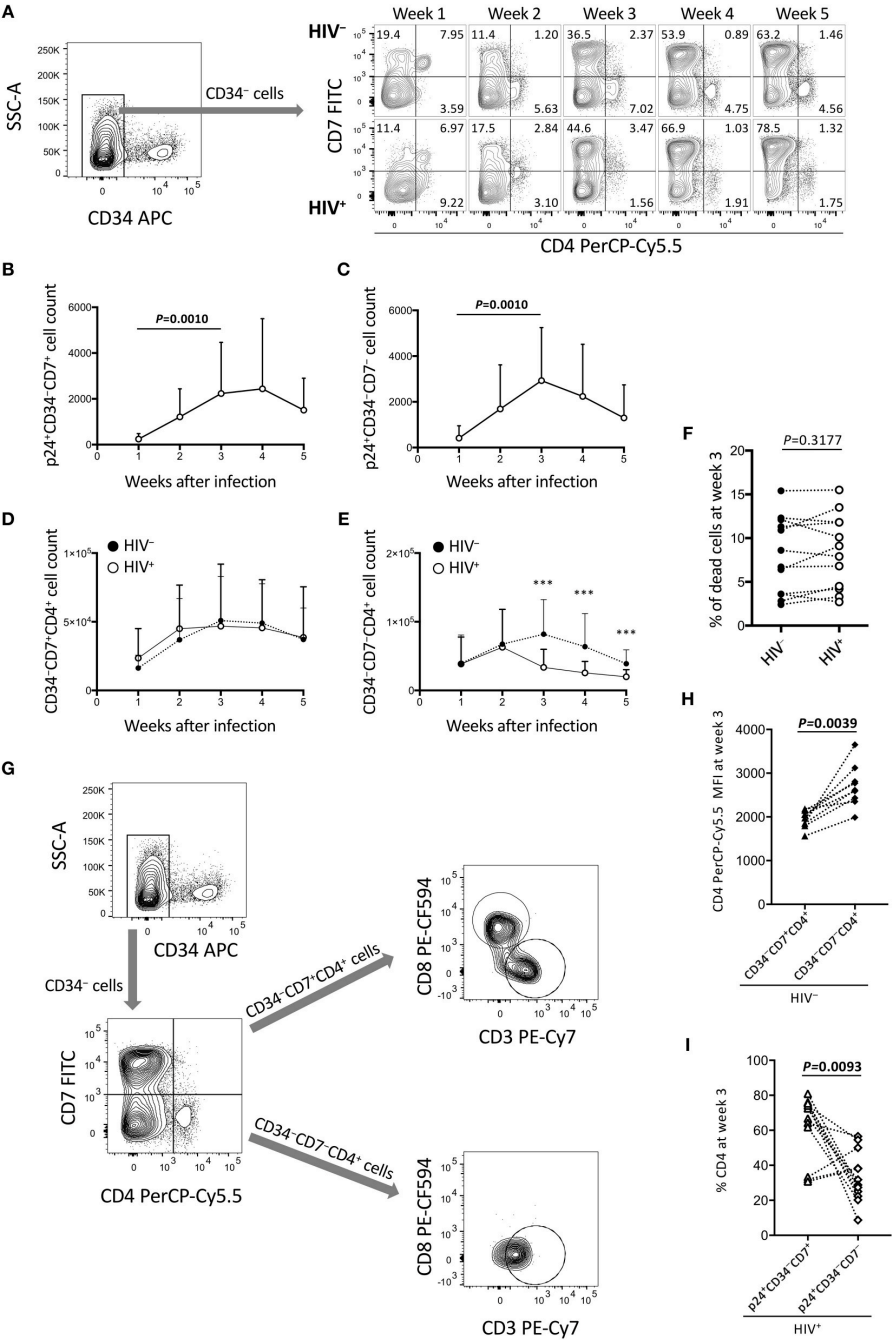
Reduction of CD34^−^CD7^−^CD4^+^ cells in the OP9-DL1 cocultures of experiment 1 (long coculture). Pre-exposure of primary cord-derived CD34^+^ cells to HIV-1 affected the dynamics of OP9-DL1 cocultured cells (*n* = 12). **(A)** Representative plots showing changes in CD7/CD4 expression patters of CD34^−^ cells in the HIV-infected cocultures at 1–5 weeks after infection. **(B)** p24^+^CD34^−^CD7^+^ cell counts. **(C)** p24^+^CD34^−^CD7^−^ cell counts. **(D,E)** Cell counts were compared between HIV^+^ and HIV^−^ cocultures for **(D)** CD34^−^CD7^+^CD4^+^ and **(E)** CD34^−^CD7^−^CD4^+^ cells. **(F)** Percentages of dead cells at week 3 post coculture were compared between HIV^+^ and HIV^−^ samples. **(G)** Flow cytometry plots showing the phenotype analysis of CD34^−^CD4^+^ cells in an OP9-DL1 coculture. Representative plots are shown. Cord-derived CD34^+^ cells were cocultured with OP9-DL1 cells for 4 weeks. Cells were collected and analyzed by flow cytometry. CD34^−^ cells were gated by CD7 and CD4 expression. CD7^+^CD4^+^ and CD7^−^CD4^+^ cells were further gated by CD8 and CD3 expression. The analysis revealed that CD7^+^CD4^+^ cells contained different subsets including CD3^−^CD4^+^CD8^+^ and CD3^+^CD4^+^CD8^−^ cells, whereas CD7^−^CD4^+^ cells were mostly CD3^+^CD4^+^CD8^−^ cells. These results indicate that CD4/CD8 double-positive thymocytes, CD7^+^ CD4 T cells, and CD7^−^ CD4 T cells were generated via coculture of cord-derived CD34^+^ and OP9-DL1 cells. **(H)** CD4 PerCP-Cy5.5 mean fluorescence intensities (MFIs) of CD34^−^CD7^+^CD4^+^ and CD34^−^CD7^−^CD4^+^ cells in uninfected coculture samples at week 3 post infection (*n* = 7). Samples stained with anti-CD4 PE-Cy7 were not included in the analysis because the different staining index with PE-Cy7 from PerCP-Cy5.5 could affect the analysis. **(I)** Percentages of CD4^+^ cells at week 3 post coculture were compared between p24^+^CD34^−^CD7^+^ and p24^+^CD34^−^CD7^−^ cells in HIV-infected coculture samples (*n* = 12). Statistical analyses were performed using the Wilcoxon matched-pairs signed rank test. ***P < 0.001.

**Figure 4:**
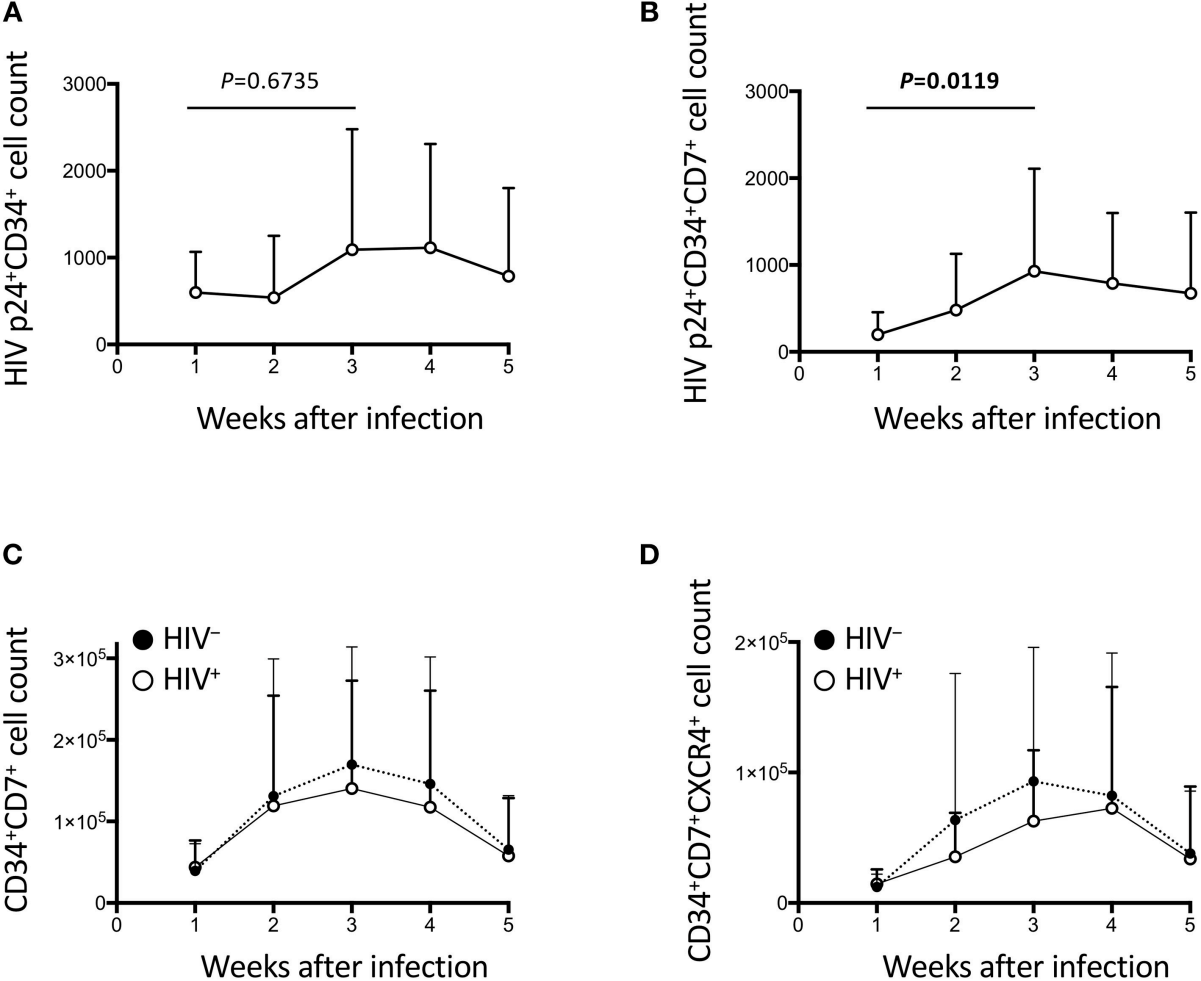
The impact of HIV on CD34^+^ cells in the OP9-DL1 cocultures of experiment 1 (long coculture). Pre-exposure of primary cord-derived CD34^+^ cells to HIV-1 affected the dynamics of CD34^+^ cells in the subsequent OP9-DL1 cocultures (*n* = 12). **(A)** HIV p24^+^CD34^+^ cell counts in HIV^+^ OP9-DL1 cocultures. **(B)** HIV p24^+^CD34^+^CD7^+^ cell counts in HIV^+^ OP9-DL1 cocultures. **(C,D)** Cell counts were compared between HIV^+^ and HIV^−^ cocultures for **(C)** CD34^+^CD7^+^ and **(D)** CD34^+^CD7^+^CXCR4^+^ cells. Statistical analyses were performed using the Wilcoxon matched-pairs signed rank test.

Two separate *in vitro* experiments were performed in the study. The data for experiment 1 (long coculture) are shown in Figures 1–4 and Figures S1–S3. To follow the long-term *in vitro* fate of HIV–pre-exposed CD34^+^ cells and derivatives for several weeks, primary human umbilical cord-derived CD34^+^ cells were infected with CXCR4-tropic HIV-1_NL4−3_ (Figure 1A). These cells were partially CD4^+^ and/or CXCR4^+^ before infection (Figure 1B), and confirmed to be partially susceptible to HIV-1_NL4−3_ infection. Cells were seeded in 48-well plates and exposed to 200 ng (p24) of HIV-1_NL4−3_. Following centrifugation and overnight incubation in the presence of HIV-1, the cells were cocultured with OP9-DL1 cells (Figure 1A). After a week of coculture, intracellular HIV-1 p24 was detected in both CD34^+^ and CD34^−^ cells (Figure 1C). HIV infection was further examined via magnetic bead separation of CD34^+^ and CD34^−^ cells followed by DNA extraction and detection of HIV-1 *gag* DNA using PCR (Figure 1D).

The HIV-1 p24 expressing cells were analyzed weekly by flow cytometry until week 5 post coculture. Total p24^+^ cell counts were calculated and shown in Figure 2A. This confirms that the HIV-infected samples displayed persistent HIV-1 p24 expression for 5 weeks. Figure 2B shows percentages of p24^+^ cells in the whole cells. These ranged from 0.1 to 1.7% of the total cocultured human cells. Viral replication was further confirmed by measuring supernatant HIV-1 p24 concentrations by ELISA (Figure 2C). A correlation was found between intracellular HIV-1 p24^+^ cell counts and supernatant HIV-1 p24 concentrations (Figure 2D). For those infected samples detecting p24^+^CD34^+^ cells (*n* = 12), the p24 MFIs of p24^+^CD34^−^ and p24^+^CD34^+^ cells were not significantly different at any time from week 1 to week 5 post infection (Figure 2E).

### The Dynamics of CD4^+^ Cells in OP9-DL1 Cocultures With HIV–Pre-Exposed CD34^+^ Cells

The long-term influence of HIV exposure of CD34^+^ cells and their T-lineage derivatives such as CD34^+^CD7^+^ lymphoid progenitors and CD4^+^ thymocytes/T cells, as well as persistent HIV replication in the cocultures, on post-coculture events was analyzed by flow cytometry weekly until week 5. CD19^+^, CD20^+^, or CD33^+^ cells were not detected in the samples tested (data not shown). HIV-infected cocultures had significantly lower whole cell counts at weeks 3, 4, and 5 post-coculture than uninfected cocultures (Figure S1A), demonstrating reduced cell growth in the presence of HIV-1. Similarly, the CD4^+^CD8^+^ and CD4^+^CD8^+^CXCR4^+^ cell counts were significantly lower in HIV^+^ cocultures than in HIV^−^ cocultures at week 3 (Figures S1B–D). Following these observations, CD34^−^ cells were gated by CD7 expression and analyzed for intracellular HIV p24 expression and differentiation to CD4^+^ cells (Figure 3). Both CD34^−^CD7^+^ and CD34^−^CD7^−^ cells, as well as both CD34^−^CD7^+^CD4^+^ and CD34^−^CD7^−^CD4^+^ cells, showed persistent p24 expression from week 1 to week 5 (Figures 3B,C, Figures S2E,F), in relation to CXCR4 expression in both CD34^−^CD7^+^CD4^+^ and CD34^−^CD7^−^CD4^+^ cells (Figure S2G). However, the CD34^−^CD7^+^CD4^+^ cell counts were not significantly different between HIV-infected and uninfected samples (Figure 3D). By contrast, there was a notable decline in CD34^−^CD7^−^CD4^+^ cell counts in HIV^+^ samples compared to those in HIV^−^ samples at week 3 (Figure 3E), which was not associated with significantly modified cell death rates by HIV exposure (Figure 3F). Further gating analysis of CD34^−^ cells revealed that the CD7^+^CD4^+^ cells contained CD4/CD8 double-positive cells and CD3^+^CD4^+^ cells, whereas the CD7^−^CD4^+^ cells were mostly CD3^+^CD4^+^ cells (Figure 3G). Similarly, CD34^−^CD7^−^CXCR4^+^ cells exhibited the greatest growth impairment following HIV-1 infection, as compared to CD34^−^CXCR4^+^ and CD34^−^CD7^+^CXCR4^+^ cells (Figure S2).

CD34^−^CD7^+^CD4^+^ and CD34^−^CD7^−^CD4^+^ cells were further characterized to clarify HIV infectivity to those subsets. For example, for those uninfected samples stained with anti-CD4 PerCP-Cy5.5 (*n* = 9), the CD4 mean fluorescence intensity (MFI) of CD34^+^CD7^+^CD4^+^ cells (mean ± SD: 1,944 ± 196) was significantly lower than that of CD34^+^CD7^−^CD4^+^ cells (mean ± SD: 2,703 ± 476) (Figure 3H). On the other hand, for all the HIV-infected samples tested (*n* = 12), the mean CD4^+^ percentage was significantly higher in p24^+^CD34^+^CD7^+^ cells (mean ± SD: 62.0 ± 18.8) than in p24^+^CD34^+^CD7^−^ cells (mean ± SD: 33.48 ± 14.6) at week 3 post infection (Figure 3I), possibly reflecting CD4 downregulation in productively infected CD34^−^CD7^−^CD4^+^ cells.

### The Dynamics of CD34^+^ Cells in OP9-DL1 Cocultures With HIV–Pre-Exposed CD34^+^ Cells

HIV-1 infection of CD34^+^ cells was also measured (Figures 4A–B, Figures S3A–D). p24^+^CD34^+^ cells failed to show significant growth by week 2 (Figure 4A), in contrast to the statistically significant growth of p24^+^ cells at this time point (Figure 2A). p24^+^CD34^+^CD7^+^ cell counts significantly increased from week 1 to week 3 (Figure 4B) in association with a low average CD34^+^CD7^+^ cell count at week 1 after infection and a rapid increase of CD34^+^CD7^+^ cell counts at weeks 2–3 (Figure 4C). Then, CD34^+^ cell dynamics in HIV^+^ samples were compared with those in HIV^−^ samples (Figures 4C,D, Figures S3E–G). There were nonsignificant differences in CD34^+^CD7^+^ cell counts between HIV^+^ and HIV^−^ cocultures (Figure 4C). The difference in the average frequencies of CD34^+^CD7^+^ cells was greatest at week 3 (Figure 4C). However, the average CD34^+^CD7^+^CXCR4^+^ cell count in HIV^+^ cocultures versus that in HIV^−^ cocultures exhibited the greatest difference at week 2 (Figure 4D). Therefore, CD34^+^CD7^+^CXCR4^+^ cells could be affected by HIV-1 earlier than the entire CD34^+^CD7^+^ cell population or cells of other phenotypes.

### HIV-1_**NL4-3**_ Infection of HSPC-Derived Cells After 4–6 Weeks of Coculture With OP9-DL1 Cells

**Figure 5:**
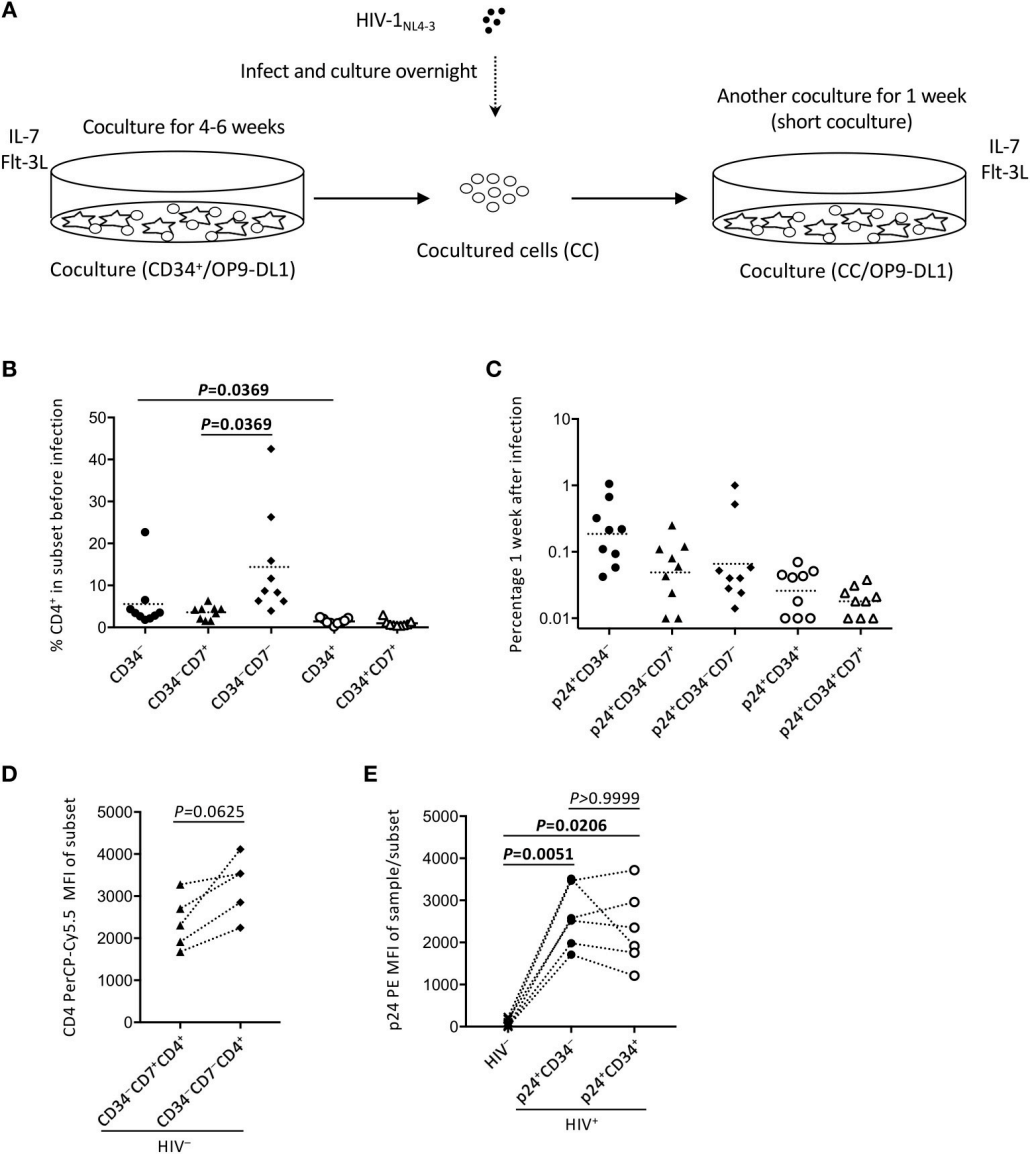
The design of experiment 2 (short coculture). **(A)** Schematic representation of experiment 2. Primary cord-derived CD34^+^ cells were cocultured with OP9-DL1 cells for 4–6 weeks. Cells were then collected and infected with HIV-1_NL4−3_. The infected cells were cocultured again with OP9-DL1 cells for 1 week, collected, and analyzed. **(B)** Percentages of CD4^+^ cells in CD34^−^, CD34^−^CD7^+^, CD34^−^CD7^−^, CD34^+^, and CD34^+^CD7^+^ subsets before infection. A multiple comparison test was performed to compare CD34^−^ and CD34^+^ cells or CD34^−^CD7^+^ and CD34^−^CD7^−^ cells. **(C)** Frequencies of HIV p24^+^CD34^−^, p24^+^CD34^−^CD7^+^, p24^+^CD34^−^CD7^−^, p24^+^CD34^+^, and p24^+^CD34^+^CD7^+^ cells measured 1 week after the second post-infection coculture (*n* = 9). **(D)** CD4-PerCP-Cy5.5 mean fluorescence intensities (MFIs) were compared between CD34^−^CD7^+^CD4^+^ cells and CD34^−^CD7^−^CD4+ cells in uninfected samples (*n* = 9). Samples stained with anti-CD4 PE-Cy7 were not included in the analysis because the different staining index with PE-Cy7 from PerCP-Cy5.5 might affect the analysis. **(E)** p24-PE mean MFIs of p24^+^CD34^−^ and p24^+^CD34^+^ cells in HIV-infected samples were compared to p24-PE MFIs of autologous uninfected samples (*n* = 6). Statistical analysis was performed using the multiple comparison test with Dunn's method.

**Figure 6:**
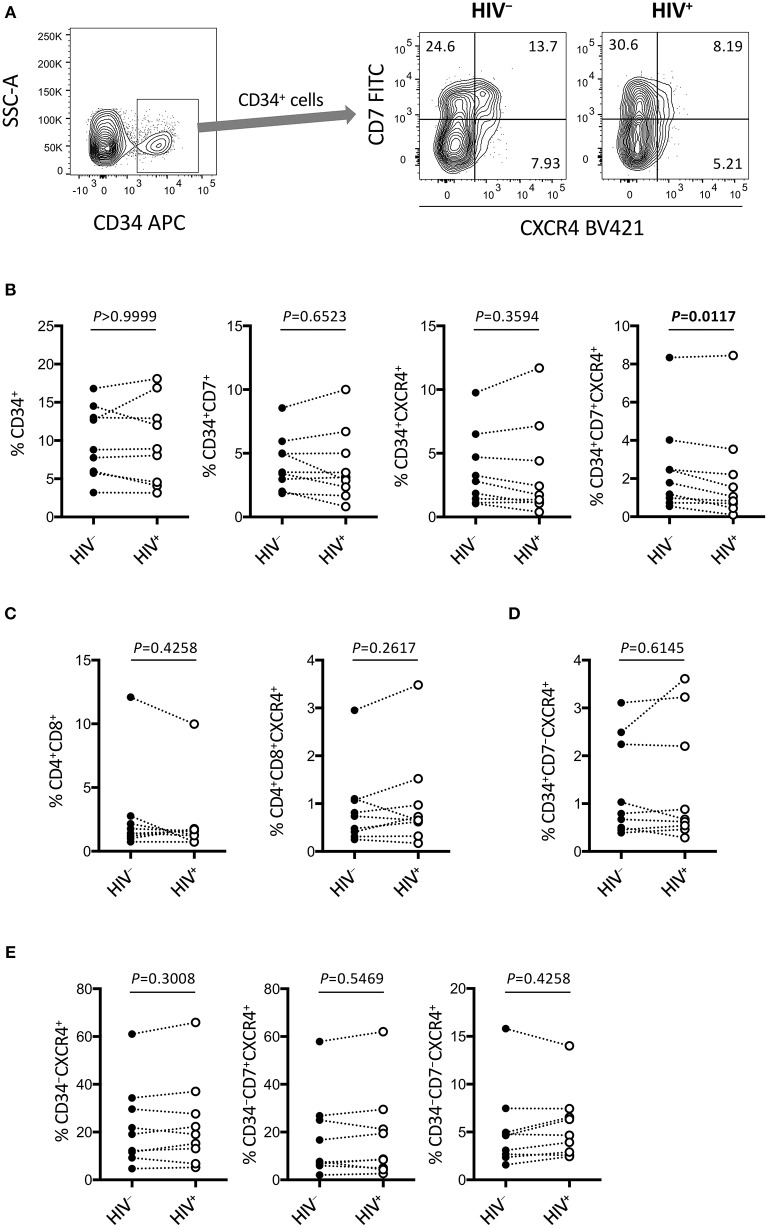
Rapid depletion of CD34^+^CD7^+^CXCR4^+^ cells in the OP9-DL1 cocultures of experiment 2 (short coculture). CD34^+^ and other subsets of cells in experiment 2 (*n* = 9) were analyzed 1 week after the second post-infection coculture. **(A)** Representative plots showing CD7/CXCR4 expression levels in CD34^+^ cells. An HIV^+^ sample is compared to its autologous uninfected counterpart. **(B–E)** Frequencies of different subsets of cells were compared between HIV^+^ and HIV^−^ samples. **(B)** CD34^+^ (left), CD34^+^CD7^+^ (middle left), CD34^+^CXCR4^+^ (middle right), and CD34^+^CD7^+^CXCR4^+^ (right) cells. **(C)** CD4^+^CD8^+^ (left) and CD4^+^CD8^+^CXCR4^+^ (right) cells. **(D)** CD34^+^CD7^−^CXCR4^+^ cells. **(E)** CD34^−^CXCR4^+^ (left), CD34^−^CD7^+^CXCR4^+^ (middle), and CD34^−^CD7^−^CXCR4^+^ (right) cells. Statistical analyses were performed using the Wilcoxon matched-pairs signed rank test.

**Figure 7:**
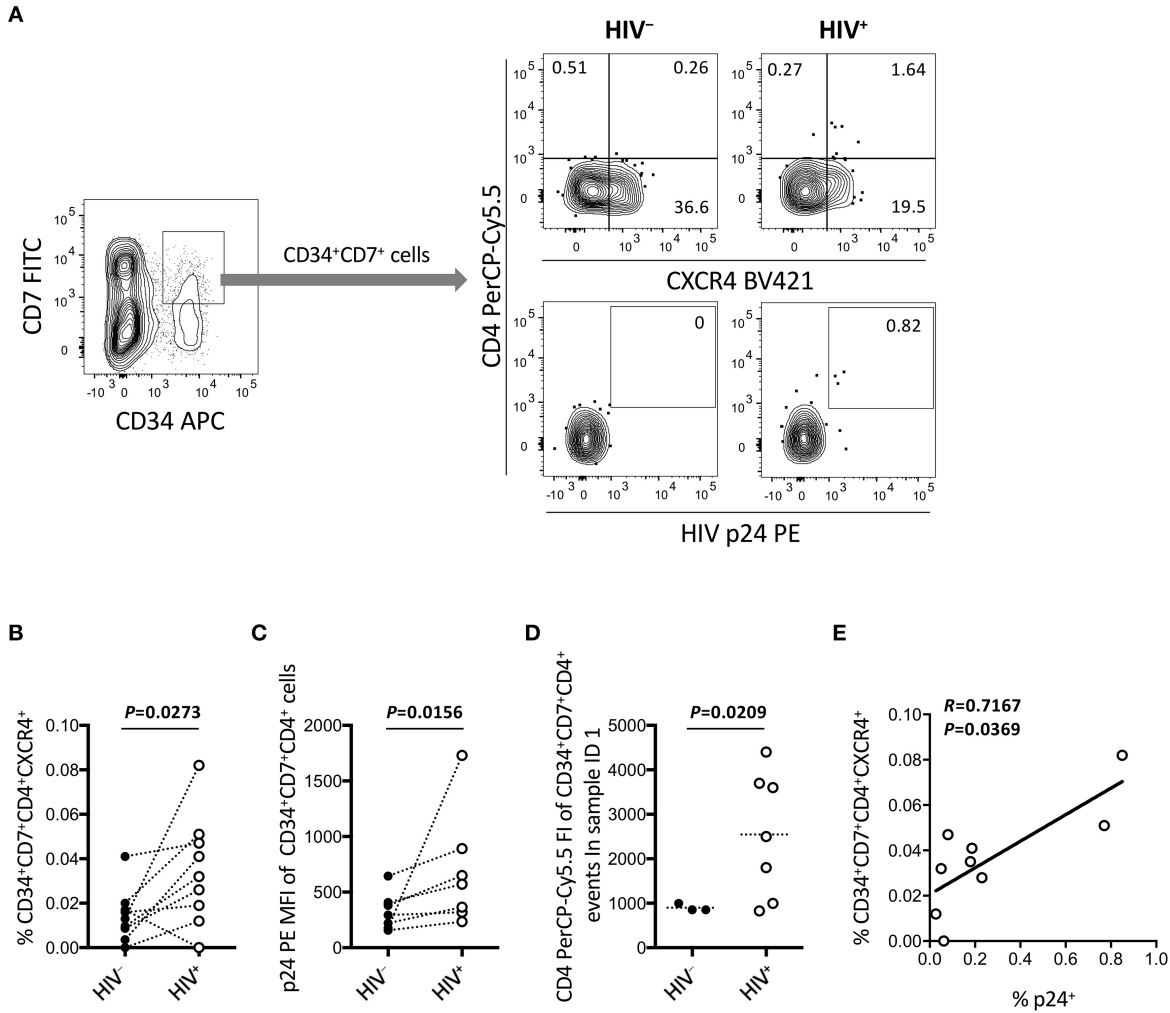
Emergence of CD34^+^CD7^+^CD4^+^CXCR4^+^ cells in the OP9-DL1 cocultures of experiment 2 (short coculture). CD34^+^CD7^+^ cells in experiment 2 (short coculture, *n* = 9) were further analyzed 1 week after the second post-infection coculture. For these analyses, gating strategies and compensation matrices were checked carefully to exclude false-positive events and avoid misinterpretation of the rare CD34^+^CD7^+^CD4^+^CXCR4^+^ events. **(A)** Representative plots showing CD4/CXCR4 and CD4/p24 expression levels in CD34^+^CD7^+^ cells. An HIV^+^ sample is compared to its autologous uninfected counterpart. **(B)** Comparison of the frequencies of CD34^+^CD7^+^CD4^+^CXCR4^+^ cells between HIV^+^ and HIV^−^ samples. **(C)** p24 mean fluorescence intensities (MFIs) of CD34^+^CD7^+^CD4^+^ cells were compared between HIV^+^ and HIV^−^ samples (*n* = 7 for HIV^+^; *n* = 3 for HIV^−^). **(D)** Fluorescence intensities (FIs) of CD34^+^CD7^+^CD4^+^ cells were compared between HIV^+^ and HIV^−^ samples using the Mann-Whitney test. **(E)** The frequencies of CD34^+^CD7^+^CD4^+^CXCR4^+^ cells were correlated with those of p24^+^ cells. Comparisons were performed using the Wilcoxon matched-pairs signed rank test. Spearman's correlation coefficient was calculated for the correlation analysis.

To better describe the effect of HIV infection on the short-term dynamics of CD34^+^ cells, experiment 2 was performed (short coculture, *n* = 12, Figure 5A), and the results are shown in Figures 5–7, Figures S4, S5, and Tables 1, 2. Briefly, coculture of OP9-DL1 cells with HIV-uninfected cord-derived CD34^+^ cells for 4–6 weeks produced a mixture of cells with different phenotypes as determined and characterized by the expression of CD34, CD4, CD8, CD7, and CXCR4 (Figures 1–4, Figures S1–S3), better resembling the *in vivo* T-lineage differentiation setting compared to experiment 1 which started with purified CD34^+^ cells cocultured with OP9-DL1 to analyze the long-term effects. CD4^+^ frequencies in CD34^+^ cells before infection were lower than those in CD34^−^ cells (Figure 5B). Cells were then harvested and infected with HIV-1_NL4−3_ following the procedures for HIV infection of CD34^+^ cells performed in experiment 1. The infected cells were cocultured again with a new OP9-DL1 monolayer and further incubated for 1 week (Figure 5A). One week after infection, HIV replication was detected in all HIV-infected samples (Figure 5C). The majority of HIV-1 p24^+^ cells were CD34^−^ (Figure 5C, Figure S4), while the majority of p24^+^CD34^+^ cells were CD7^+^ (Figure 5C, Table 1). For those uninfected samples stained with anti-CD4 PerCP-Cy5.5 (*n* = 5), the CD4 MFI of CD34^+^CD7^+^CD4^+^ cells (mean ± SD: 2,375 ± 638) tended to be lower than that of CD34^+^CD7^−^CD4^+^ cells (mean ± SD: 3,257 ± 720) (Figure 5D). For those infected samples detecting p24^+^CD34^+^ cells (*n* = 6), the p24 MFIs of p24^+^CD34^−^ (mean ± SD: 2,628 ± 743) and p24^+^CD34^+^ (mean ± SD: 2,319 ± 903) cells were not significantly different (Figure 5E).

**Table 1:** Mean cell counts (*n* = 4) in different HIV p24^+^ subsets of OP9-DL1 cocultured cells.

**Subset**	**Cell count in infected samples (mean ± SD, *n* = 4)**	**Percentage in the mean whole-cell count**
Whole	390,000 ± 155,000	100
p24^+^	547 ± 258	0.140
p24^+^CD34^−^CD7^+^	326 ± 242	0.084
p24^+^CD34^−^CD7^−^	124 ± 56	0.032
p24^+^CD34^+^	97 ± 42	0.025
p24^+^CD34^+^CD7^+^	80 ± 31	0.021

*The whole-cell count data were obtained using four of nine samples from experiment 2 (short coculture), in which cord-derived CD34^+^ cells were first cocultured with OP9-DL1 cells for 4–6 weeks, infected with HIV-1_NL4-3_, cocultured again for 1 week and analyzed by flow cytometry. The percentages in the mean whole-cell count are also shown, and they are comparable to those presented in Figure 5C. SD, standard deviation*.

**Table 2:** Mean cell counts (*n* = 4) in different subsets of OP9-DL1–cocultured cells.

**Subset**	**Cell count (mean ± SD**, ***n*** **= 4)**		**% $\frac{\left(Infected\;-\;Uninfected\right)}{Uninfected}$**
	**Uninfected**	**Infected**
Whole	400,000 ± 169,000	390,000 ± 155,000	−2.5
CD4^+^CD8^+^	5,790 ± 3,510	4,920 ± 2,360	**−15.0**
CD4^+^CD8^+^CXCR4^+^	3,290 ± 1,760	3,050 ± 1,840	−7.3
CD34^+^	32,800 ± 18,200	32,100 ± 21,400	−2.3
CD34^+^CD7^+^	16,700 ± 9,500	16,100 ± 12,100	−3.6
CD34^+^CXCR4^+^	18,300 ± 11,000	18,300 ± 14,700	−0.1
CD34^+^CD7^+^CXCR4^+^	12,100 ± 7,800	11,900 ± 11,100	−2.0
CD34^+^CD7^−^CXCR4^+^	6,150 ± 3,870	6,380 ± 3,990	+3.7
CD34^+^CD7^+^CD4^+^	129 ± 42	178 ± 74	**+38.0**
CD34^−^CXCR4^+^	148,000 ± 164,000	157,000 ± 167,000	+5.8
CD34^−^CD7^+^CXCR4^+^	131,000 ± 161,000	139,000 ± 162,000	+5.6
CD34^−^CD7^−^CXCR4^+^	16,900 ± 7,500	18,300 ± 8,000	+7.8

*The whole-cell count data were obtained using four of nine samples from experiment 2 (short coculture), in which cord-derived CD34^+^ cells were first cocultured with OP9-DL1 for 4–6 weeks, infected with HIV-1_NL4-3_, cocultured again for 1 week and analyzed by flow cytometry. Cell counts in HIV-infected samples were compared with those in uninfected control samples. Percent changes in the subset cell counts following HIV infection were calculated, as shown in the right column. Percent changes of >10% are shown in bold. SD, standard deviation*.

### Partial Loss of CD34^+^CD7^+^CXCR4^+^ Cells After HIV-1_**NL4-3**_ Infection of OP9-DL1–Cocultured Cells

The phenotypes of the cells in experiment 2 (short coculture) were analyzed 1 week after infection (Figure 6, Table 2). The whole cell counts were obtained using parts of the tested batches, with no significant differences noted between HIV^+^ and HIV^−^ samples (*n* = 4, *p* = 0.5000, Table 2). The gating strategy based on the expression of CD34, CD7, and CXCR4 is shown in Figure 6A. The frequencies of CD34^+^ and CD34^+^CD7^+^ cells (Figure 6B) were not significantly different between HIV^+^ and HIV^−^ cocultures. Regarding CXCR4 expression levels in different subsets, the frequencies of CD34^+^CD7^+^CXCR4^+^ cells in HIV^+^ samples (mean 2.08 ± 2.56%) were significantly reduced compared to those in HIV^−^ samples (mean 2.49 ± 2.42%) (Figure 6B right), regardless of the degree of infection (data not shown). This may be in accordance with the experiment 1 results in which the growth of CD34^+^CD7^+^CXCR4^+^ cells slowed earlier (at week 2, Figure 4D) than those of CD34^+^CD7^−^CD4^−^ cells (at week 3, Figure 3E). The frequencies of CD4^+^CD8^+^ cells (Figure 6C), CD34^+^CD7^−^CXCR4^+^ cells (Figure 6D), and subsets of CD34^−^ cells (Figure 6E) were not significantly different between HIV^+^ and HIV^−^ cocultures.

### Increased Number of CD34^+^CD7^+^CXCR4^+^CD4^+^ Cells After HIV-1_**NL4-3**_ Infection of OP9-DL1–Cocultured Cells

Figure 6 indicates the distinct dynamics of CD34^+^CD7^+^CXCR4^+^ cells in the presence of HIV-1. For increased clarity, CD34^+^CD7^+^ cells were further analyzed for CD4 expression (Figure 7 and Figure S5). Surprisingly, HIV-infected samples had higher frequencies of CD34^+^CD7^+^CD4^+^CXCR4^+^ cells than uninfected samples (Figures 7A,B), although caution is necessary in interpreting the data because of the rarity of CD34^+^CD7^+^CD4^hi^ cells in each HIV^+^ sample (Figure 7A). Further analyses were performed to carefully characterize the rare CD34^+^CD7^+^CD4^hi^ events. These events were found to be partially HIV p24^+^ after carefully adjusting the compensation matrices of the flow cytometry data (Figures 7A,C). One of nine batches was selected for further analyses of CD34^+^CD7^+^CD4^+^ cells. Both the frequencies and CD4 fluorescence intensities of CD34^+^CD7^+^CD4^+^ cells were higher in HIV^+^ samples than in HIV^−^ samples (Figure 7D), implying the emergence of CD34^+^CD7^+^CD4^hi^ cells. The frequencies of CD34^+^CD7^+^CD4^+^CXCR4^+^ cells were correlated with those of p24^+^ cells (Figure 7E). A separate staining of an experiment 2 sample with Annexin V was performed at week 1 after infection to check for apoptosis. There was no increase in Annexin V reactivity of CD34^+^CD4^+^ cells in HIV^+^ samples compared to those in HIV^−^ samples (data not shown). Results from further analyses of CD34^+^ cells expressing CD4 are presented in Figure S5.

### Potential Influence of HIV-1 on the Differentiation Rate of CD34^+^CD7^+^Lymphoid Progenitor Cells Predicted by Theoretical Modeling of the OP9-DL1 Coculture Results

A newly developed theoretical model is proposed to generate hypotheses that may explain the *in vitro* OP9-DL1 coculture results and thus be tested in future studies (Figure S6). Briefly, the dynamics of CD34^+^CD7^−^ cells (*S*), CD34^+^CD7^+^ cells (*P*), CD7^+^CD4^+^ cells (*Y*), and CD7^−^CD4^+^ cells (*M*) in the presence or absence of HIV (*V*) were modeled by considering competition among the cells for the CXCR4/SDF-1 signals, and adjusting the parameters to fit the data shown in Figures 1–4 and Figures S1–S3 (Figures S6A,B). Sample outputs were calculated (Figure S6C). These analyses indicated that δ_2_ or κ_2_, which represent the differentiation rate or death rate of CD34^+^CD7^+^ cells (*P*), respectively, could be defined as a function of viral load (*V*) to replicate the quick depletion of CD34^+^CD7^+^ cells (Figure S6C).

## Discussion

The dynamics of CD34^+^ cells in HIV-infected individuals has been of great interest. For example, there has been a debate regarding whether HSPCs comprise an unignorable viral reservoir that remains undifferentiated for a long time and prevents complete cure (68). HIV might also modify the turnover of HSPCs through infection and depletion of CD4^+^ cells, leading to the common manifestation of bone marrow abnormalities (7). Some patients fail to exhibit CD4^+^ T-cell recovery even after effective antiretroviral therapy, and such immunological nonresponders can be associated with immune activation and/or bone marrow impairment (69, 70). These problems are addressed in the present study employing the OP9-DL1 coculture system, which enables *in vitro* follow-up of the early events in T-cell differentiation, such as lymphoid progenitor cell generation, that normally occur in the bone marrow, and also CD4^+^ thymocyte differentiation in the thymus.

The events observed in the cocultures of OP9-DL1 and human CD34^+^ cells are likely to involve interactions between SDF-1 and CXCR4, as mouse SDF-1 expressed by OP9-DL1 cells has high amino acid sequence identity with human SDF-1 (71). This is in accordance with previous reports illustrating that the SDF-1/CXCR4 pair is crucially involved in the homing and repopulation of HSPCs in specific bone marrow niches (72) and also in the entire T-cell developmental process in the thymus (57, 73). Regarding HIV susceptibility of HSPCs and thymocytes, they express CXCR4, but their CCR5 expression is limited (74). HIV thus utilizes CXCR4 when it infects multipotent progenitor cells (11), although some natural variants of SDF-1 may interfere with the interaction of HIV-1 Env with CXCR4 (75). As such, CXCR4-tropic HIV-1 strains might contribute to pathogenesis by interfering with hematopoiesis and/or lymphopoiesis (76–78), but the underlying mechanisms are yet to be clarified.

In most HIV-1 positive patients including adults and children, CCR5-tropic HIV-1 replicates first, while CXCR4-tropic HIV-1 appears later in the course of disease (79). However, a substantial proportion of children show rapid disease progression and mortality (80), which is associated with CXCR4 tropism (81). This may be partly because of the active role of thymus in children in the development of mature T lymphocytes, while the thymic microenvironment further enhances the CXCR4 tropism of thymocytes (82) Recent reports showed, using humanized mouse models, that infection with CCR5-tropic HIV-1 strains may result in depletion of bone marrow CD34^+^ cells, which was dependent on the presence of plasmacytoid dendritic cells (pDCs) (9) or associated with the expression of CXCR4 (83). As such, CCR5-troipic HIV-1 infection alone may be sufficient for causing bone marrow abnormalities such as in anemia and pancytopenia. On the other hand, the present study utilizing coculture of HIV-infected CD34^+^ cells and OP9-DL1 showed that CXCR4-tropic HIV-1 infection may also cause rapid depletion of CD34^+^CD7^+^CXCR4^+^ cells. Therefore, both CCR5-tropic viruses and CXCR4-tropic viruses may cause the impairment of the host's hematopoietic potential. This could explain the association of the early appearance of CXCR4-tropic virus with enhanced CD4^+^ cell depletion and progression to AIDS (51).

The present results provide insights into the impact of HIV-1 on T-lineage differentiation of hematopoietic progenitor cells. In experiment 1 (long coculture, Figures 1–4), HIV-infected samples exhibited similar CD4^+^ cell production rates as uninfected samples at weeks 1 and 2 but reduced CD4^+^ cell production rates from week 3 to week 5 (Figure 3). This was most clearly observed with the dynamics of CD34^−^CD7^−^CD4^+^ cells (Figure 3E) that were mostly CD3^+^ T cells (Figure 3G) and expressing higher levels of CD4 than CD34^−^CD7^+^CD4^+^ cells (Figure 3H). It is still unclear how the reduction of cell growth at week 3 after infection was so accurately reproduced in the 12 samples tested (Figure 3E, individual data not shown). However, in the subsequent analyses of CD34^+^ cells, the growth rates of CD34^+^CD7^+^CXCR4^+^ cells tended to fall as early as week 2 (Figure 4D). This was observed in 7/12 samples tested (data not shown), and the decline occurred earlier than the reduction in CD34^−^CD7^−^CD4^+^ cell counts (week 3, Figure 3E). Because CD34^+^CD7^+^ cells represent lymphoid progenitor cells (21, 25), it is possible that they could be partly involved in defining the production rates of CD4^+^ cells.

Subsequently, experiment 2 (short coculture, Figures 5–7) was conducted to examine the effects of HIV-1 on the short-term dynamics of T-lineage cells including CD34^+^ progenitors. HIV-1 infection resulted in significantly decreased frequency of CD34^+^CD7^+^CXCR4^+^ cells 1 week after infection (Figure 6B right), which is consistent with the results of experiment 1 (Figure 4D). This study is limited in that the fate of the lost CD34^+^CD7^+^CXCR4^+^ cells in the HIV^+^ cocultures remains unknown. The differentiation capacity of the remaining CD34^+^CD7^+^CXCR4^+^ cells also remains to be tested. The CD34^+^CD7^+^ progenitors were further analyzed to better understand their association with HIV-1 infection. Interestingly, the frequencies of CD34^+^CD7^+^CD4^+^ cells were elevated in HIV-infected samples compared to those in uninfected samples (Figure 7). In addition, those small numbers of CD34^+^CD7^+^CD4^+^ cells were found to be mostly CXCR4^+^ and partially HIV p24^+^ (Figures 7A,C). It may be tempting to interpret the results as CD4 upregulation following HIV infection, thereby driving the differentiation of T-lineage precursor cells. However, caution may be necessary because exclusion of possible CD34 upregulation in the infected CD4^+^ cells was not confirmed in this study (Figure S5), although the flow cytometry data did not indicate correlations between HIV p24 expression and CD34 upregulation in CD34^low^ cells (data not shown). The rarity of CD34^+^CD7^+^CD4^hi^ cells in each HIV^+^ sample is also noted (Figure 7A), which might be insufficient to fully explain the observed reduction of CD34^+^CD7^+^CXCR4^+^ cell counts in HIV-infected samples (Figure 6A,B). Further studies may be designed to test shorter coculture periods than 1 week and/or investigate the association of HIV-1 infection with factors that regulate the expression of CD3, CD4, CD7, and CD34. Such analyses will also help better clarify the debate on the issues of CD34^+^ viral reservoirs (68).

**Figure 8:**
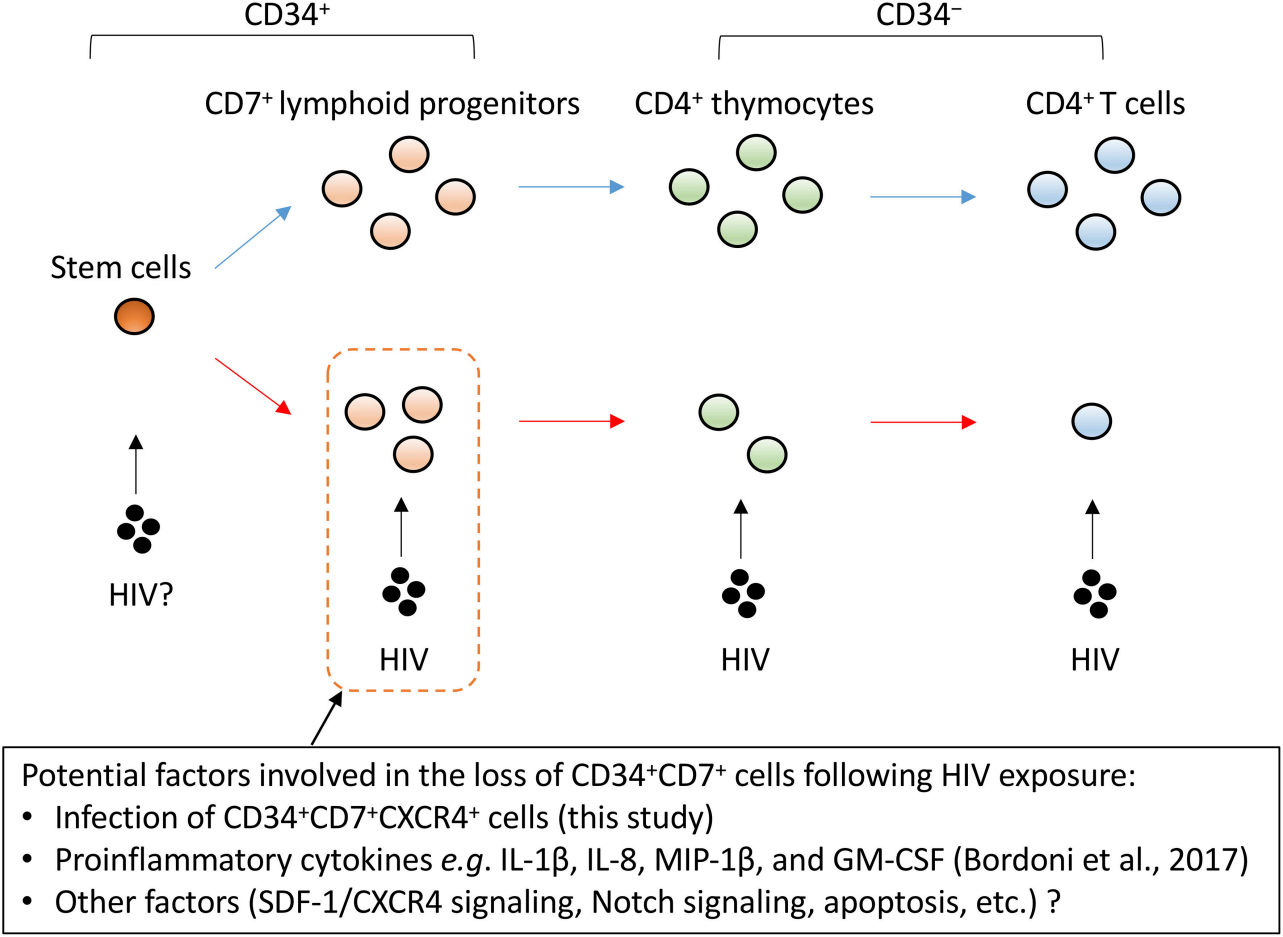
A schematic representation of factors potentially influencing the decrement of CD4^+^ T cells via CD34^+^CD7^+^ lymphoid progenitor cell dynamics. CD4^+^ thymocytes and T cells are highly susceptible to HIV infection. However, the decrease of CD34^+^CD7^+^ lymphoid progenitor counts occurs early and precedes the decrease of thymocyte and T-cell counts. Although the mechanisms for the decrease of CD34^+^ lymphoid progenitors are unclear, this decline may limit the production of thymocytes and T cells.

Possible mechanisms for the rapid depletion of CD34^+^CD7^+^ cells in the presence of sustained HIV replication (Figures 4D, 6) were investigated for generating hypotheses to be tested in future studies by modeling the present study results (Figure S6). The model was first developed to reflect competition of cells for the CXCR4/SDF-1 homing signals (Figures S6A,B). However, this didn't result in replication of the preceding depletion of CD34^+^CD7^+^ cells to other cell types (Figure S6C middle), which is consistent with the small frequencies of direct productive HIV infection of CD34^+^CD7^+^CXCR4^+^ cells (Figure 5C). The model was then modified to also reflect potential influence of HIV upon the differentiation rate (δ_2_) or death rate (κ_2_) of CD34^+^CD7^+^ cells in an indirect manner. This allowed the model to better fit the *in vitro* OP9-DL1 coculture data (Figure S6C right). These modeling results might indicate partial impact of the direct infection of CD34^+^CD7^+^ cells with HIV-1 (Figure 5C) (84), and hence raise questions regarding additional molecular mechanisms underlying the loss (differentiation or death) of CD34^+^CD7^+^CXCR4^+^ cells, such as the Notch signaling pathway, SDF-1/CXCR4 signaling pathway, or inflammatory signals as described above. It may be crucial to better understand the impact of HIV on these programs in lymphopoiesis and T-cell development. For example, the involvement of HIV has been indicated in the pathogenesis of nephropathy and neuronal disorders in infected patients via activation of the Notch signaling pathway (85, 86). Regarding inflammatory signals, a recent study reported that bone marrow CD34^+^ progenitor cells from HIV-infected patients exhibit impaired T-cell differentiation potential (87), which was related to proinflammatory cytokines such as IL-1β, IL-8, MIP-1β, and GM-CSF. Cytokine analyses may be applicable to the coculture assays established in the present study (Figure 8). These collectively would lead to further clarification of the contribution of indirect mechanisms relative to the extent of direct HIV infection of lymphoid progenitors.

Similarly, one may test whether differentiation rates of CD34^+^ progenitors in the HIV-1 presence can be modified by blocking the CXCR4/SDF-1 pathway (Figure 8). Regarding the point, the increased plasma levels of SDF-1 in late-stage HIV-infected patients was reported previously (88). A recent study using a humanized mouse model further described depletion of bone marrow CD34^+^ cells following CCR5-tropic HIV-1 infection in a CXCR4-associated manner (83). In light of this, it may be worth testing *in vitro* whether CCR5-tropic HIV-1 strains can cause depletion of CD34^+^CD7^+^CXCR4^+^ lymphoid progenitors. The dynamics of CD34^+^ cells following their infection with HIV-1 may be indeed a complex phenomenon, depending on the proportion of target cells for HIV-1, the expression of restriction factors in those target cells, and the microenvironment including cytokines and chemokines, and also cell turnover. The *in vitro* OP9-DL1 coculture model and the phenotypic analysis established in this study will allow further close analyses to clarify the mechanisms for impaired T-lineage generation in HIV-infected settings.

The findings from this study may highlight the potential of anti-HIV treatments such as gene therapy using CD34^+^ HSPCs followed by transplantation because in this manner, all hematopoietic events in the host can be protected against HIV infection by the gene products even in the absence of effective immune responses (89). It is important to keep in mind that although some researchers may consider CCR5 a reasonable target for knockout or knockdown to prevent infection by CCR5-tropic HIV strains, the manipulation of CXCR4 expression levels on HSPCs should be considered more carefully because of the essential biological functions of the molecule (90). Therefore, instead of modulating CXCR4 expression, anti-HIV modalities targeting an HIV gene or component should probably protect hematopoietic cells including T-lineage cells and CD34^+^ cells from CXCR4-tropic HIV infection (83).

In summary, this study established an *in vitro* OP9-DL1 coculture model to analyze T-lineage differentiation from HSPCs to CD4^+^ T cells in the presence of HIV-1. The results of the long/short coculture of human CD34^+^ cells and derivatives with OP9-DL1 cells in the presence of HIV-1 indicate that the dynamics of CD34^+^CD7^+^ lymphoid progenitors may be affected in a CXCR4-associated manner and more quickly than CD34^−^CD4^+^ thymocytes and T cells despite the lower susceptibility of CD34^+^CD7^+^ cells to HIV-1 infection as suggested by their lower CD4^+^ frequencies. These observations should be further verified *in vivo* in the future. The newly developed OP9-DL1/HIV-1 model may allow further studies to elucidate the underling mechanisms. In addition, the possible reduction of CD34^+^CD7^+^CXCR4^+^ cell counts preceding the reduction of CD34^−^CD4^+^ cell growth in the HIV^+^ cocultures might illustrate the potential mechanism by which changes in progenitor cell pools including reduced sizes may contribute to decelerated production of T cells in HIV-infected patients (Figure 8). This could occur in combination with different mechanisms of CD4^+^ T-cell depletion including direct cytopathic effects, apoptosis and antigen-specific immunological mechanisms (91–93).

## Author Contributions

TT performed experiments, analyzed data and wrote the manuscript.

### Conflict of Interest Statement

The author declares that the research was conducted in the absence of any commercial or financial relationships that could be construed as a potential conflict of interest.

## References

[1] Parinitha S Kulkarni M. Haematological changes in HIV infection with correlation to CD4 cell count. Australas Med J. (2012) 5:157–62. 10.4066/AMJ.2012.100 22952560 PMC3433730

[2] Corbeau P Reynes J. Immune reconstitution under antiretroviral therapy: the new challenge in HIV-1 infection. Blood (2011) 117:5582–90. 10.1182/blood-2010-12-322453 21403129

[3] Blom B Epeldegui M Uittenbogaart CH Factors contributing to HIV-1 induced pathogenesis in the human thymus. In: Chang TL-Y, editor. HIV-Host Interactions. Rijeka: InTech (2011). p. 149–82.

[4] Ye P Kirschner DE Kourtis AP. The thymus during HIV disease: role in pathogenesis and in immune recovery. Curr HIV Res. (2004) 2:177–83. 10.2174/1570162043484898 15078181

[5] Knutsen AP Roodman ST Freeman JJ Mueller KR Bouhasin JD. Inhibition of thymopoiesis of CD34^+^ cell maturation by HIV-1 in an *in vitro* CD34^+^ cell and thymic epithelial organ culture model. Stem Cells (1999) 17:327–38. 10.1002/stem.170327 10606161

[6] Alexaki A Wigdahl B. HIV-1 infection of bone marrow hematopoietic progenitor cells and their role in trafficking and viral dissemination. PLoS Pathog. (2008) 4:e1000215. 10.1371/journal.ppat.1000215 19112504 PMC2603331

[7] Dhurve SA Dhurve AS. Bone marrow abnormalities in HIV disease. Mediterr J Hematol Infect Dis. (2013) 5:e2013033. 10.4084/mjhid.2013.033 23795271 PMC3684351

[8] Banda NK Simon GR Sipple JD Terrell KL Archer P Shpall EJ. Depletion of CD34^+^ CD4^+^ cells in bone marrow from HIV-1-infected individuals. Biol Blood Marrow Transplant. (1999) 5:162–72. 10.1053/bbmt.1999.v5.pm10392962 10392962

[9] Sebastian NT Zaikos TD Terry V Taschuk F Mcnamara LA Onafuwa-Nuga A. CD4 is expressed on a heterogeneous subset of hematopoietic progenitors, which persistently harbor CXCR4 and CCR5-tropic HIV proviral genomes *in vivo*. PLoS Pathog. (2017) 13:e1006509. 10.1371/journal.ppat.1006509 28732051 PMC5540617

[10] Carter CC Onafuwa-Nuga A Mcnamara LA Riddell JT Bixby D Savona MR. HIV-1 infects multipotent progenitor cells causing cell death and establishing latent cellular reservoirs. Nat Med. (2010) 16:446–51. 10.1038/nm.2109 20208541 PMC2892382

[11] Carter CC Mcnamara LA Onafuwa-Nuga A Shackleton M Riddell JT Bixby D. HIV-1 utilizes the CXCR4 chemokine receptor to infect multipotent hematopoietic stem and progenitor cells. Cell Host Microbe (2011) 9:223–34. 10.1016/j.chom.2011.02.005 21402361 PMC3102232

[12] Griffin DO Goff SP. HIV-1 Is Restricted prior to integration of viral DNA in primary cord-derived human CD34^+^ cells. J Virol. (2015) 89:8096–100. 10.1128/JVI.01044-15 25995256 PMC4505644

[13] Tsukamoto T Okada S. The use of RetroNectin in studies requiring *in vitro* HIV-1 infection of human hematopoietic stem/progenitor cells. J Virol Methods (2017) 248:234–7. 10.1016/j.jviromet.2017.08.003 28789988

[14] Nakano T Kodama H Honjo T. Generation of lymphohematopoietic cells from embryonic stem cells in culture. Science (1994) 265:1098–101. 10.1126/science.8066449 8066449

[15] Schmitt TM De Pooter RF Gronski MA Cho SK Ohashi PS Zuniga-Pflucker JC. Induction of T cell development and establishment of T cell competence from embryonic stem cells differentiated *in vitro*. Nat Immunol. (2004) 5:410–7. 10.1038/ni1055 15034575

[16] De Smedt M Hoebeke I Plum J Human bone marrow CD34^+^ progenitor cells mature to T cells on OP9-DL1 stromal cell line without thymus microenvironment. Blood Cells Mol Dis. (2004) 33:227–32. 10.1016/j.bcmd.2004.08.007 15528136

[17] Oberlin E Amara A Bachelerie F Bessia C Virelizier JL Arenzana-Seisdedos F. The CXC chemokine SDF-1 is the ligand for LESTR/fusin and prevents infection by T-cell-line-adapted HIV-1. Nature (1996) 382:833–5. 10.1038/382833a0 8752281

[18] Janas ML Varano G Gudmundsson K Noda M Nagasawa T Turner M. Thymic development beyond beta-selection requires phosphatidylinositol 3-kinase activation by CXCR4. J Exp Med. (2010) 207:247–61. 10.1084/jem.20091430 20038597 PMC2812547

[19] Marechal V Arenzana-Seisdedos F Heard JM Schwartz O. Opposite effects of SDF-1 on human immunodeficiency virus type 1 replication. J Virol. (1999) 73:3608–15. 10196252 10.1128/jvi.73.5.3608-3615.1999 PMC104135

[20] Mohtashami M Shah DK Nakase H Kianizad K Petrie HT Zuniga-Pflucker JC. Direct comparison of Dll1- and Dll4-mediated Notch activation levels shows differential lymphomyeloid lineage commitment outcomes. J Immunol. (2010) 185:867–76. 10.4049/jimmunol.1000782 20548034

[21] Awong G Herer E Surh CD Dick JE La Motte-Mohs RN Zuniga-Pflucker JC. Characterization *in vitro* and engraftment potential *in vivo* of human progenitor T cells generated from hematopoietic stem cells. Blood (2009) 114:972–82. 10.1182/blood-2008-10-187013 19491395

[22] Awong G Singh J Mohtashami M Malm M La Motte-Mohs RN Benveniste PM. Human proT-cells generated *in vitro* facilitate hematopoietic stem cell-derived T-lymphopoiesis *in vivo* and restore thymic architecture. Blood (2013) 122:4210–9. 10.1182/blood-2012-12-472803 24215033 PMC5527400

[23] Kondo M Weissman IL Akashi K. Identification of clonogenic common lymphoid progenitors in mouse bone marrow. Cell (1997) 91:661–72. 10.1016/S0092-8674(00)80453-5 9393859

[24] Prohaska SS Scherer DC Weissman IL Kondo M. Developmental plasticity of lymphoid progenitors. Semin Immunol. (2002) 14:377–84. 10.1016/S1044532302000726 12457610

[25] Hoebeke I De Smedt M Stolz F Pike-Overzet K Staal FJ Plum J T-, B- and NK-lymphoid, but not myeloid cells arise from human CD34(+)CD38(-)CD7(+) common lymphoid progenitors expressing lymphoid-specific genes. Leukemia (2007) 21:311–9. 10.1038/sj.leu.2404488 17170726

[26] Bender JG Unverzagt KL Walker DE Lee W Van Epps DE Smith DH. Identification and comparison of CD34-positive cells and their subpopulations from normal peripheral blood and bone marrow using multicolor flow cytometry. Blood (1991) 77:2591–6. 1710512

[27] Anderson G Moore NC Owen JJ Jenkinson EJ. Cellular interactions in thymocyte development. Annu Rev Immunol. (1996) 14:73–99. 10.1146/annurev.immunol.14.1.73 8717508

[28] Lam GK Liao HX Xue Y Alam SM Scearce RM Kaufman RE. Expression of the CD7 ligand K-12 in human thymic epithelial cells: regulation by IFN-gamma. J Clin Immunol. (2005) 25:41–9. 10.1007/s10875-005-0356-5 15742156

[29] Shimizu Y Van Seventer GA Ennis E Newman W Horgan KJ Shaw S. Crosslinking of the T cell-specific accessory molecules CD7 and CD28 modulates T cell adhesion. J Exp Med. (1992) 175:577–82. 10.1084/jem.175.2.577 1370688 PMC2119108

[30] Aandahl EM Sandberg JK Beckerman KP Tasken K Moretto WJ Nixon DF. CD7 is a differentiation marker that identifies multiple CD8 T cell effector subsets. J Immunol. (2003) 170:2349–55. 10.4049/jimmunol.170.5.2349 12594257

[31] Aandahl EM Quigley MF Moretto WJ Moll M Gonzalez VD Sonnerborg A. Expansion of CD7(low) and CD7(negative) CD8 T-cell effector subsets in HIV-1 infection: correlation with antigenic load and reversion by antiretroviral treatment. Blood (2004) 104:3672–8. 10.1182/blood-2004-07-2540 15308569

[32] Kukel S Reinhold U Oltermann I Kreysel HW. Progressive increase of CD7- T cells in human blood lymphocytes with ageing. Clin Exp Immunol. (1994) 98:163–8. 10.1111/j.1365-2249.1994.tb06624.x 7523007 PMC1534167

[33] Weiss RA. HIV receptors and the pathogenesis of AIDS. Science (1996) 272:1885–6. 10.1126/science.272.5270.1885 8658158

[34] Grivel JC Margolis LB. CCR5- and CXCR4-tropic HIV-1 are equally cytopathic for their T-cell targets in human lymphoid tissue. Nat Med. (1999) 5:344–6. 10.1038/6565 10086394

[35] Schnittman SM Lane HC Greenhouse J Justement JS Baseler M Fauci AS. Preferential infection of CD4+ memory T cells by human immunodeficiency virus type 1: evidence for a role in the selective T-cell functional defects observed in infected individuals. Proc Natl Acad Sci USA. (1990) 87:6058–62. 10.1073/pnas.87.16.6058 2385584 PMC54471

[36] Okoye AA Picker LJ. CD4(+) T-cell depletion in HIV infection: mechanisms of immunological failure. Immunol Rev. (2013) 254:54–64. 10.1111/imr.12066 23772614 PMC3729334

[37] Berges BK Akkina SR Folkvord JM Connick E Akkina R Mucosal transmission of R5 and X4 tropic HIV-1 via vaginal and rectal routes in humanized Rag2-/- gammac -/- (RAG-hu) mice. Virology (2008) 373:342–51. 10.1016/j.virol.2007.11.020 18207484 PMC3092740

[38] Richman DD Bozzette SA. The impact of the syncytium-inducing phenotype of human immunodeficiency virus on disease progression. J Infect Dis. (1994) 169:968–74. 10.1093/infdis/169.5.968 7909549

[39] Shankarappa R Margolick JB Gange SJ Rodrigo AG Upchurch D Farzadegan H. Consistent viral evolutionary changes associated with the progression of human immunodeficiency virus type 1 infection. J Virol. (1999) 73:10489–502. 10559367 10.1128/jvi.73.12.10489-10502.1999 PMC113104

[40] Schuitemaker H Koot M Kootstra NA Dercksen MW De Goede RE Van Steenwijk RP. Biological phenotype of human immunodeficiency virus type 1 clones at different stages of infection: progression of disease is associated with a shift from monocytotropic to T-cell-tropic virus population. J Virol. (1992) 66:1354–60. 1738194 10.1128/jvi.66.3.1354-1360.1992 PMC240857

[41] Karlsson A Parsmyr K Sandstrom E Fenyo EM Albert J. MT-2 cell tropism as prognostic marker for disease progression in human immunodeficiency virus type 1 infection. J Clin Microbiol. (1994) 32:364–70. 7908672 10.1128/jcm.32.2.364-370.1994 PMC263037

[42] Connor RI Sheridan KE Ceradini D Choe S Landau NR. Change in coreceptor use correlates with disease progression in HIV-1–infected individuals. J Exp Med. (1997) 185:621–8. 10.1084/jem.185.4.621 9034141 PMC2196142

[43] Scarlatti G Tresoldi E Bjorndal A Fredriksson R Colognesi C Deng HK. In vivo evolution of HIV-1 co-receptor usage and sensitivity to chemokine-mediated suppression. Nat Med. (1997) 3:1259–65. 10.1038/nm1197-1259 9359702

[44] Yu XF Wang Z Vlahov D Markham RB Farzadegan H Margolick JB. Infection with dual-tropic human immunodeficiency virus type 1 variants associated with rapid total T cell decline and disease progression in injection drug users. J Infect Dis. (1998) 178:388–96. 10.1086/515646 9697718

[45] Daar ES Kesler KL Petropoulos CJ Huang W Bates M Lail AE. Baseline HIV type 1 coreceptor tropism predicts disease progression. Clin Infect Dis. (2007) 45:643–9. 10.1086/520650 17683002

[46] Shepherd JC Jacobson LP Qiao W Jamieson BD Phair JP Piazza P. Emergence and persistence of CXCR4-tropic HIV-1 in a population of men from the multicenter AIDS cohort study. J Infect Dis. (2008) 198:1104–12. 10.1086/591623 18783316 PMC2753263

[47] Waters L Mandalia S Randell P Wildfire A Gazzard B Moyle G. The impact of HIV tropism on decreases in CD4 cell count, clinical progression, and subsequent response to a first antiretroviral therapy regimen. Clin Infect Dis. (2008) 46:1617–23. 10.1086/587660 18419499

[48] Weiser B Philpott S Klimkait T Burger H Kitchen C Burgisser P. HIV-1 coreceptor usage and CXCR4-specific viral load predict clinical disease progression during combination antiretroviral therapy. Aids (2008) 22:469–79. 10.1097/QAD.0b013e3282f4196c 18301059

[49] Zhou Y Shen L Yang HC Siliciano RF. Preferential cytolysis of peripheral memory CD4+ T cells by *in vitro* X4-tropic human immunodeficiency virus type 1 infection before the completion of reverse transcription. J Virol. (2008) 82:9154–63. 10.1128/JVI.00773-08 18596085 PMC2546913

[50] Sheppard HW Celum C Michael N L O'brien S Dean M Carrington M. HIV-1 infection in individuals with the CCR5-Delta32/Delta32 genotype: acquisition of syncytium-inducing virus at seroconversion. J Acquir Immune Defic Syndr. (2002) 29:307–13. 10.1097/00126334-200203010-00013 11873082

[51] Koot M Keet IP Vos AH De Goede RE Roos MT Coutinho RA. Prognostic value of HIV-1 syncytium-inducing phenotype for rate of CD4+ cell depletion and progression to AIDS. Ann Intern Med. (1993) 118:681–8. 10.7326/0003-4819-118-9-199305010-00004 8096374

[52] Blaak H Van't Wout AB Brouwer M Hooibrink B Hovenkamp E Schuitemaker H. *In vivo* HIV-1 infection of CD45RA(+)CD4(+) T cells is established primarily by syncytium-inducing variants and correlates with the rate of CD4(+) T cell decline. Proc Natl Acad Sci USA. (2000) 97:1269–74. 10.1073/pnas.97.3.1269 10655520 PMC15592

[53] Valenzuela-Fernandez A Palanche T Amara A Magerus A Altmeyer R Delaunay T. Optimal inhibition of X4 HIV isolates by the CXC chemokine stromal cell-derived factor 1 alpha requires interaction with cell surface heparan sulfate proteoglycans. J Biol Chem. (2001) 276:26550–8. 10.1074/jbc.M100411200 11352904

[54] Kucia M Jankowski K Reca R Wysoczynski M Bandura L Allendorf DJ. CXCR4-SDF-1 signalling, locomotion, chemotaxis and adhesion. J Mol Histol. (2004) 35:233–45. 10.1023/B:HIJO.0000032355.66152.b8 15339043

[55] Lapidot T Kollet O. The essential roles of the chemokine SDF-1 and its receptor CXCR4 in human stem cell homing and repopulation of transplanted immune-deficient NOD/SCID and NOD/SCID/B2m(null) mice. Leukemia (2002) 16:1992–2003. 10.1038/sj.leu.2402684 12357350

[56] Kucia M Reca R Miekus K Wanzeck J Wojakowski W Janowska-Wieczorek A. Trafficking of normal stem cells and metastasis of cancer stem cells involve similar mechanisms: pivotal role of the SDF-1-CXCR4 axis. Stem Cells (2005) 23:879–94. 10.1634/stemcells.2004-0342 15888687

[57] Plotkin J Prockop SE Lepique A Petrie HT. Critical role for CXCR4 signaling in progenitor localization and T cell differentiation in the postnatal thymus. J Immunol. (2003) 171:4521–7. 10.4049/jimmunol.171.9.4521 14568925

[58] Berkowitz RD Beckerman KP Schall TJ Mccune JM. CXCR4 and CCR5 expression delineates targets for HIV-1 disruption of T cell differentiation. J Immunol. (1998) 161:3702–10. 9759895

[59] Suzuki H Motohara M Miyake A Ibuki K Fukazawa Y Inaba K. Intrathymic effect of acute pathogenic SHIV infection on T-lineage cells in newborn macaques. Microbiol Immunol. (2005) 49:667–79. 10.1111/j.1348-0421.2005.tb03646.x 16034211

[60] Marsden MD Kovochich M Suree N Shimizu S Mehta R Cortado R. HIV latency in the humanized BLT mouse. J Virol. (2012) 86:339–47. 10.1128/JVI.06366-11 22072769 PMC3255908

[61] Dudek TE Allen TM. HIV-specific CD8(+) T-cell immunity in humanized bone marrow-liver-thymus mice. J Infect Dis. (2013) 208 (Suppl. 2):S150–4. 10.1093/infdis/jit320 24151322 PMC3807972

[62] Adachi A Gendelman HE Koenig S Folks T Willey R Rabson A. Production of acquired immunodeficiency syndrome-associated retrovirus in human and nonhuman cells transfected with an infectious molecular clone. J Virol. (1986) 59:284–91. 3016298 10.1128/jvi.59.2.284-291.1986 PMC253077

[63] Holmes R Zuniga-Pflucker JC The OP9-DL1 system: generation of T-lymphocytes from embryonic or hematopoietic stem cells *in vitro*. Cold Spring Harb Protoc. (2009) 2009:pdb prot5156 10.1101/pdb.prot5156 20147086

[64] Lamoreaux L Roederer M Koup R. Intracellular cytokine optimization and standard operating procedure. Nat Protoc. (2006) 1:1507–16. 10.1038/nprot.2006.268 17406442

[65] Suzuki K Shijuuku T Fukamachi T Zaunders J Guillemin G Cooper D. Prolonged transcriptional silencing and CpG methylation induced by siRNAs targeted to the HIV-1 promoter region. J RNAi Gene Silencing (2005) 1:66–78. 10.1038/mtna.2014.67 19771207 PMC2737205

[66] Abkowitz JL Golinelli D Harrison DE Guttorp P. *In vivo* kinetics of murine hemopoietic stem cells. Blood (2000) 96:3399–405. 11071634

[67] Catlin SN Busque L Gale RE Guttorp P Abkowitz JL. The replication rate of human hematopoietic stem cells *in vivo*. Blood (2011) 117:4460–6. 10.1182/blood-2010-08-303537 21343613 PMC3099568

[68] Pace M O'doherty U. Hematopoietic stem cells and HIV infection. J Infect Dis. (2013) 207:1790–2. 10.1093/infdis/jit120 23554379 PMC3654756

[69] Hunt PW Martin JN Sinclair E Bredt B Hagos E Lampiris H. T cell activation is associated with lower CD4+ T cell gains in human immunodeficiency virus-infected patients with sustained viral suppression during antiretroviral therapy. J Infect Dis. (2003) 187:1534–43. 10.1086/374786 12721933

[70] Isgro A Leti W De Santis W Marziali M Esposito A Fimiani C. Altered clonogenic capability and stromal cell function characterize bone marrow of HIV-infected subjects with low CD4+ T cell counts despite viral suppression during HAART. Clin Infect Dis. (2008) 46:1902–10. 10.1086/588480 18462177

[71] Shirozu M Nakano T Inazawa J Tashiro K Tada H Shinohara T. Structure and chromosomal localization of the human stromal cell-derived factor 1 (SDF1) gene. Genomics (1995) 28:495–500. 10.1006/geno.1995.1180 7490086

[72] Moll NM Ransohoff RM. CXCL12 and CXCR4 in bone marrow physiology. Expert Rev Hematol. (2010) 3:315–22. 10.1586/ehm.10.16 21082982

[73] Petrie HT. Cell migration and the control of post-natal T-cell lymphopoiesis in the thymus. Nat Rev Immunol. (2003) 3:859–66. 10.1038/nri1223 14668802

[74] Nixon CC Vatakis DN Reichelderfer SN Dixit D Kim SG Uittenbogaart CH. HIV-1 infection of hematopoietic progenitor cells in vivo in humanized mice. Blood (2013) 122:2195–204. 10.1182/blood-2013-04-496950 23886835 PMC3785119

[75] Winkler C Modi W Smith MW Nelson GW Wu X Carrington M. Genetic restriction of AIDS pathogenesis by an SDF-1 chemokine gene variant. ALIVE study, hemophilia growth and development study (HGDS), multicenter AIDS cohort study (MACS), multicenter hemophilia cohort study (MHCS), San Francisco city cohort (SFCC). Science (1998) 279:389–93. 10.1126/science.279.5349.389 9430590

[76] Ho Tsong Fang R Colantonio AD Uittenbogaart CH The role of the thymus in HIV infection: a 10 year perspective. AIDS (2008) 22:171–84. 10.1097/QAD.0b013e3282f2589b 18097219

[77] Akkina R. New insights into HIV impact on hematopoiesis. Blood (2013) 122:2144–6. 10.1182/blood-2013-08-518274 24072846

[78] Bordoni V Casetti R Viola D Abbate I Rozera G Sacchi A. Early ART in primary HIV infection may also preserve lymphopoiesis capability in circulating haematopoietic progenitor cells: a case report. J Antimicrob Chemother. (2015) 70:1598–600. 10.1093/jac/dku559 25604747

[79] Mariani SA Brigida I Kajaste-Rudnitski A Ghezzi S Rocchi A Plebani A HIV-1 envelope-dependent restriction of CXCR4-using viruses in child but not adult untransformed CD4+ T-lymphocyte lines. Blood (2012) 119:2013–23. 10.1182/blood-2010-12-325308 22234680

[80] Reyes RA Canfield DR Esser U Adamson LA Brown CR Cheng-Mayer C. Induction of simian AIDS in infant rhesus macaques infected with CCR5- or CXCR4-utilizing Simian-human immunodeficiency viruses is associated with distinct lesions of the thymus. J Virol. (2004) 78:2121–30. 10.1128/JVI.78.4.2121-2130.2004 14747577 PMC369416

[81] Almeida FJ Zaparoli MS Moreira DH Cavalcanti Jde S Rodrigues R Berezin EN. Association of X4 tropism with disease progression in antiretroviral-treated children and adolescents living with HIV/AIDS in Sao Paulo, Brazil. Braz J Infect Dis. (2014) 18:300–7. 10.1016/j.bjid.2013.10.002 24275366 PMC9427470

[82] Schmitt N Chene L Boutolleau D Nugeyre MT Guillemard E Versmisse P. Positive regulation of CXCR4 expression and signaling by interleukin-7 in CD4+ mature thymocytes correlates with their capacity to favor human immunodeficiency X4 virus replication. J Virol. (2003) 77:5784–93. 10.1128/JVI.77.10.5784-5793.2003 12719571 PMC154045

[83] Tsukamoto T. Transcriptional gene silencing limits CXCR4-associated depletion of bone marrow CD34^+^ cells in HIV-1 infection. AIDS (2018) 32:1737–47. 10.1097/QAD.0000000000001882 29762163

[84] Lee B Doranz BJ Ratajczak MZ Doms RW. An intricate Web: chemokine receptors, HIV-1 and hematopoiesis. Stem Cells (1998) 16:79–88. 10.1002/stem.160079 9554031

[85] Sharma M Callen S Zhang D Singhal PC Vanden Heuvel GB Buch S. Activation of notch signaling pathway in HIV-associated nephropathy. AIDS (2010) 24:2161–70. 10.1097/QAD.0b013e32833dbc31 20706108 PMC3086691

[86] Fan Y Gao X Chen J Liu Y He JJ. HIV tat impairs neurogenesis through functioning as a notch ligand and activation of notch signaling pathway. J Neurosci. (2016) 36:11362–73. 10.1523/JNEUROSCI.1208-16.2016 27807176 PMC5148248

[87] Bordoni V Bibas M Viola D Sacchi A Cimini E Tumino N. Bone marrow CD34(+) progenitor cells from HIV-infected patients show an impaired T cell differentiation potential related to proinflammatory cytokines. AIDS Res Hum Retroviruses (2017) 33:590–6. 10.1089/aid.2016.0195 28125903

[88] Ikegawa M Yuan J Matsumoto K Herrmann S Iwamoto A Nakamura T. Elevated plasma stromal cell-derived factor 1 protein level in the progression of HIV type 1 infection/AIDS. AIDS Res Hum Retroviruses (2001) 17:587–95. 10.1089/088922201300119680 11375054

[89] Savkovic B Nichols J Birkett D Applegate T Ledger S Symonds G. A quantitative comparison of anti-HIV gene therapy delivered to hematopoietic stem cells versus CD4+ T cells. PLoS Comput Biol. (2014) 10:e1003681. 10.1371/journal.pcbi.1003681 24945407 PMC4063676

[90] Liu Y Zhou J Pan JA Mabiala P Guo D. A novel approach to block HIV-1 coreceptor CXCR4 in non-toxic manner. Mol Biotechnol. (2014) 56:890–902. 10.1007/s12033-014-9768-7 24845753

[91] Su L Kaneshima H Bonyhadi M Salimi S Kraft D Rabin L. HIV-1-induced thymocyte depletion is associated with indirect cytopathogenicity and infection of progenitor cells *in vivo*. Immunity (1995) 2:25–36. 10.1016/1074-7613(95)90076-4 7600300

[92] Costin JM. Cytopathic mechanisms of HIV-1. Virol J. (2007) 4:100. 10.1186/1743-422X-4-100 17945027 PMC2174939

[93] Tsukamoto T Yamamoto H Okada S Matano T. Recursion-based depletion of human immunodeficiency virus-specific naive CD4(+) T cells may facilitate persistent viral replication and chronic viraemia leading to acquired immunodeficiency syndrome. Med Hypotheses (2016) 94:81–5. 10.1016/j.mehy.2016.06.024 27515208

