# Supplementary material for: HIV Impacts CD34+ Progenitors Involved in T-Cell Differentiation During Coculture With Mouse Stromal OP9-DL1 Cells
Source: Front Immunol. 2019 Jan 29;10:81. doi: 10.3389/fimmu.2019.00081 (PMC6361802; doi:10.3389/fimmu.2019.00081)
Supplement: Supplementary file 1 [file Data_Sheet_1.PDF]

Supplemental information

Figures S1-S6

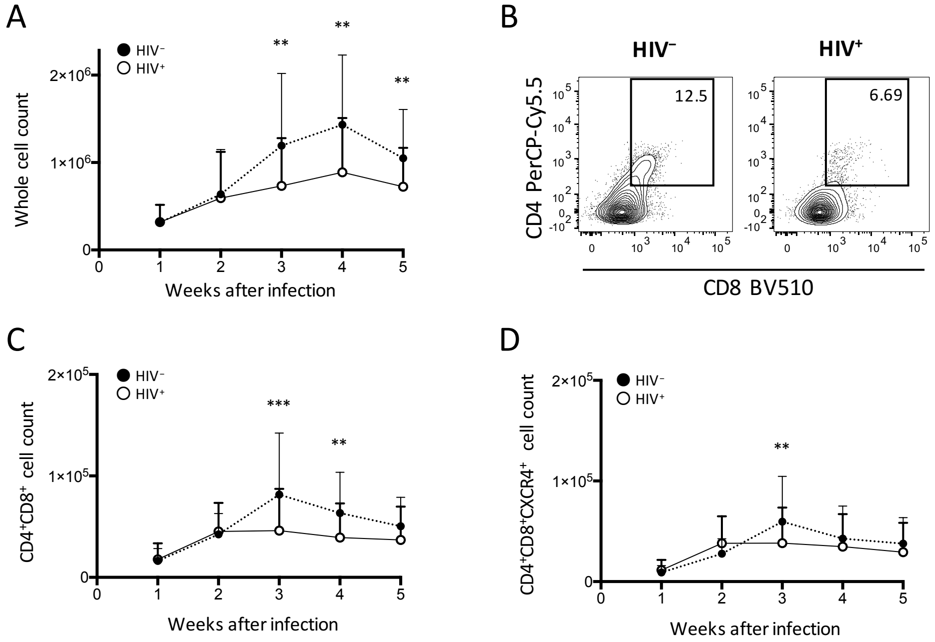

Figure S1. Pre-exposure of primary cord-derived CD34$^+$ cells to HIV-1 affected the dynamics of OP9-DL1 cocultured cells. (**A**) Whole cell counts were compared between HIV$^+$ and HIV$^-$ cocultures. (**B**) Representative plots for samples displaying reduced frequencies of CD4$^+$CD8$^+$ cells 3–5 weeks after HIV pre-exposure of primary CD34$^+$ cells and coculture. The plots were selected from week 4 samples. (**C**–**D**) Cell counts were compared between HIV$^+$ and HIV$^-$ cocultures for (**C**) CD4$^+$CD8$^+$ and (**D**) CD4$^+$CD8$^+$CXCR4$^+$ cells. Statistical analyses were performed using the Wilcoxon matched-pairs signed rank test. **: $P < 0.01$, ***: $P < 0.001$.

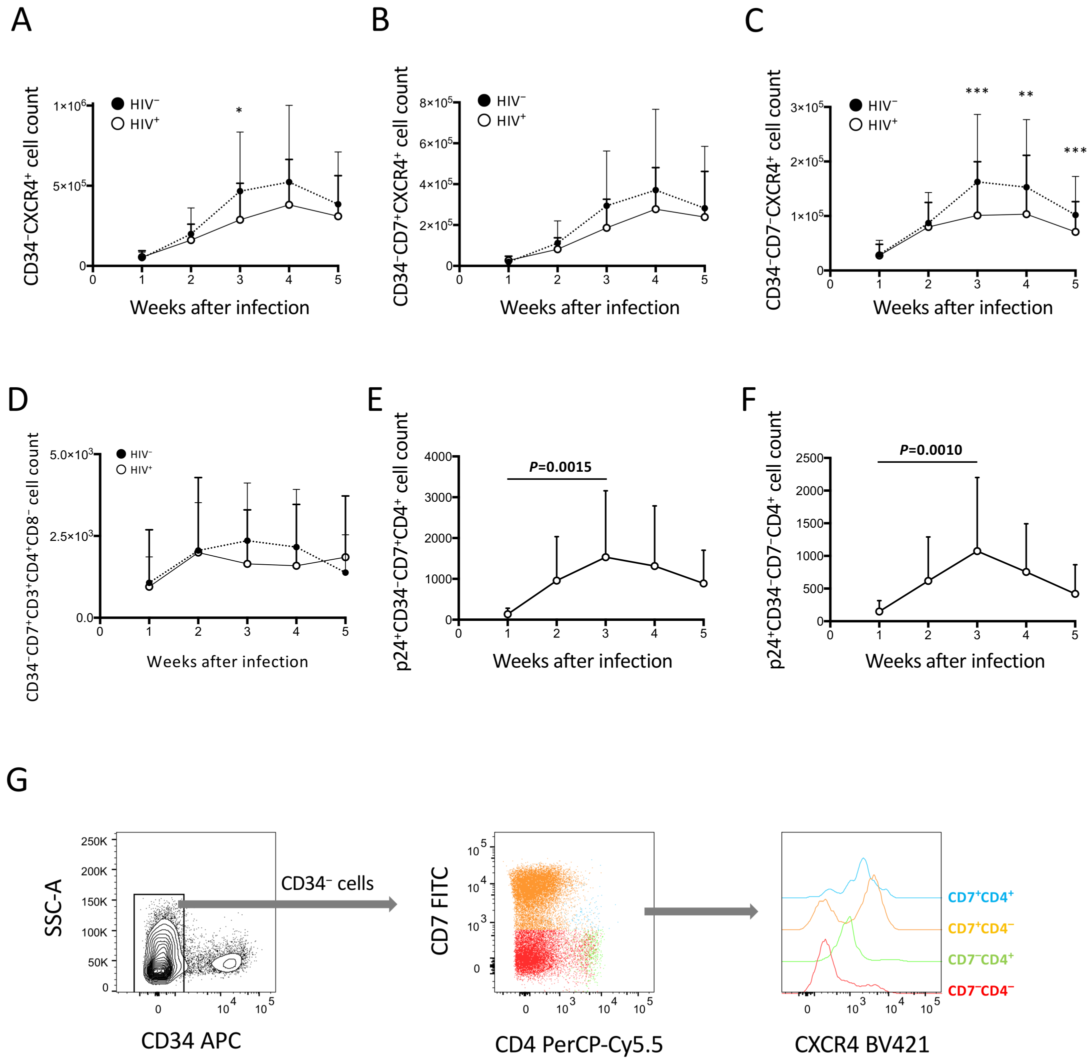

Figure S2. Phenotypes of CD34$^-$ cells in the OP9-DL1 cocultures were analyzed weekly for 5 weeks after HIV-1 infection. (**A-D**) Cell counts were compared with those in uninfected samples (n = 12). (**A**) CD34$^-$CXCR4$^+$ cell counts. (**B**) CD34$^-$CD7$^+$CXCR4$^+$ cell counts. (**C**) CD34$^-$CD7$^-$CXCR4$^+$ cell counts. (**D**) CD34$^-$CD7$^+$CD3$^+$CD4$^+$CD8$^-$ cell counts. (**E**) p24$^+$CD34$^-$CD7$^+$CD4$^+$ cell counts at weeks 1–5 post-infection. (**F**) p24$^+$CD34$^-$CD7$^+$CD4$^+$ cell counts at weeks 1–5 post-infection. (**G**) Representative plots showing the CXCR4 expression levels in different subsets (CD7$^+$CD4$^+$, CD7$^+$CD4$^-$, CD7$^-$CD4$^+$, and CD7$^-$CD4$^-$) of CD34$^-$ cells after coculture with OP9-DL1 for 4 weeks without HIV infection. Comparisons were performed using the Wilcoxon matched-pairs signed rank test. *: $P < 0.05$, **: $P < 0.01$, ***: $P < 0.001$.

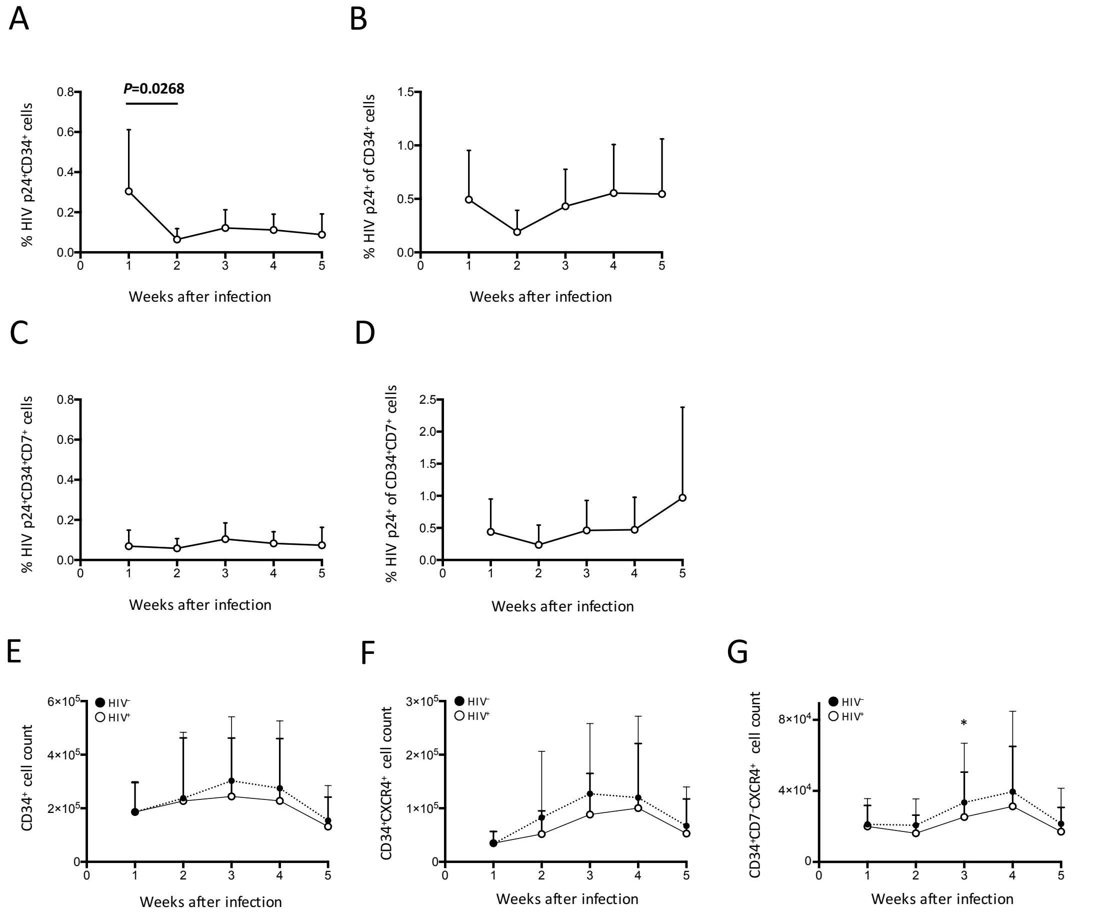

Figure S3. (**A**–**D**) HIV p24$^+$ subset dynamics were analyzed. (**A**) Percent p24$^+$CD34$^+$ cells. (**B**) Percent p24$^+$ cells among CD34$^+$ cells. (**C**) Percent p24$^+$CD34$^+$CD7$^+$ cells. (**D**) Percent p24$^+$ cells among CD34$^+$CD7$^+$ cells. (**E**–**G**) CD34$^+$ subset dynamics were analyzed and compared between HIV$^+$ and HIV$^-$ samples. (**E**) CD34$^+$ cell counts. (**F**) CD34$^+$CXCR4$^+$ cell counts. (**G**) CD34$^+$CD7$^-$CXCR4$^+$ cell counts.

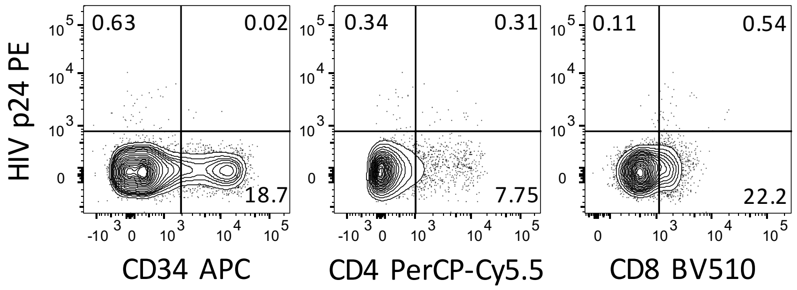

Figure S4. Experiment 2 (short coculture, n = 9) was performed to further analyze the dynamics of $CD34^+$ cells in the presence of HIV-1. Representative plots showing HIV $p24^+$ cells 1 week after infection. The majority of $p24^+$ cells were $CD34^-$ (left). HIV replication may be causing CD4 downregulation (middle). Frequencies of $CD8^+$ cells in $p24^+$ cells were variable among the samples (right).

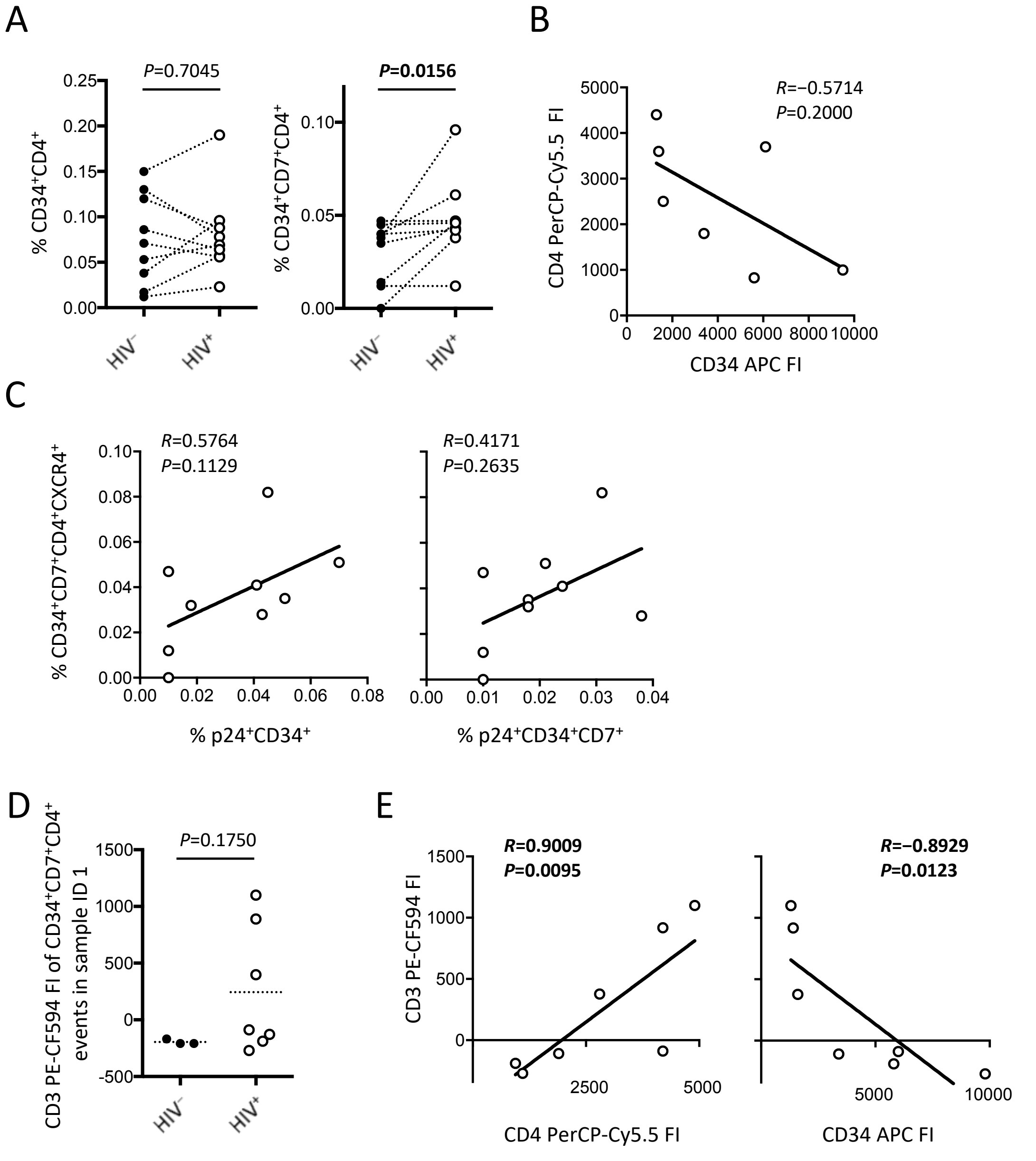

Figure S5. CD34$^+$CD7$^+$CD4$^+$ cells were further analyzed. (**A**) Comparison of CD34$^+$CD4$^+$ (left) and CD34$^+$CD7$^+$CD4$^+$ (right) frequencies between HIV$^+$ and HIV$^-$ samples (n = 9). (**B**) Correlation between the CD4 and CD34 FI of CD34$^+$CD7$^+$CD4$^+$ cells in the HIV$^+$ sample of ID 1 were analyzed (n = 7). (**C**) CD34$^+$CD7$^+$CD4$^+$CXCR4$^+$ cells were tested for correlations with the frequencies of p24$^+$CD34$^+$ (left) and p24$^+$CD34$^+$CD7$^+$ (right) cells (n = 9). (**D**) Comparison of CD3 FIs between HIV-infected and uninfected samples (n = 7 for HIV$^+$; n = 3 for HIV$^-$). (**E**) Correlation analysis between CD3 FIs and CD4 (left) or CD34 (right) FIs (n = 7). Comparisons were made using the Wilcoxon matched-pairs signed rank test unless otherwise noted. Spearman's correlation coefficients were calculated for correlation analyses.

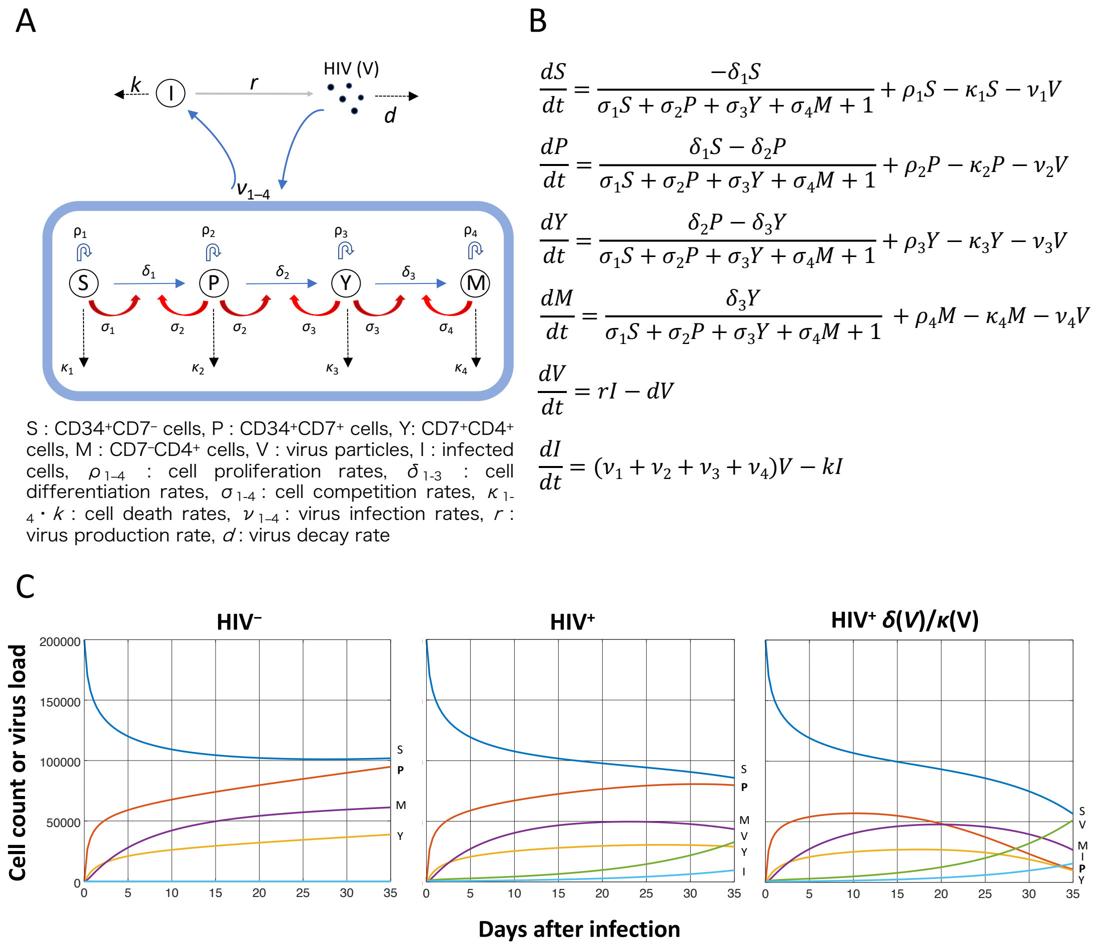

$$\frac{dS}{dt} = \frac{-\delta_1 S}{\sigma_1 S + \sigma_2 P + \sigma_3 Y + \sigma_4 M + 1} + \rho_1 S - \kappa_1 S - \nu_1 V$$

$$\frac{dP}{dt} = \frac{\delta_1 S - \delta_2 P}{\sigma_1 S + \sigma_2 P + \sigma_3 Y + \sigma_4 M + 1} + \rho_2 P - \kappa_2 P - \nu_2 V$$

$$\frac{dY}{dt} = \frac{\delta_2 P - \delta_3 Y}{\sigma_1 S + \sigma_2 P + \sigma_3 Y + \sigma_4 M + 1} + \rho_3 Y - \kappa_3 Y - \nu_3 V$$

$$\frac{dM}{dt} = \frac{\delta_3 Y}{\sigma_1 S + \sigma_2 P + \sigma_3 Y + \sigma_4 M + 1} + \rho_4 M - \kappa_4 M - \nu_4 V$$

$$\frac{dV}{dt} = rI - dV$$

$$\frac{dI}{dt} = (\nu_1 + \nu_2 + \nu_3 + \nu_4)V - kI$$

Figure S6. A theoretical model to analyze the dynamics of cells involved in T-cell differentiation in the presence of sustained HIV replication. The model was constructed from scratch to roughly fit the present data shown in Figs. 1–4 (experiment 1), with an assumption that $CXCR4^{+}$ cells may possibly compete with each other for the use of CXCR4/SDF-1 signaling pathway. (**A**) A schematic description of the model. (**B**) A mathematical description of the model. (**C**) Sample outputs of the model run with the parameters set to roughly fit the data in Figs 1-3. "HIV^{-}" (left) represents results in the absence of HIV. "HIV^{+}" (center) represents results in the presence of HIV. "HIV^{+} $\delta(V)/\kappa(V)$" (right) is similar to "HIV^{+}" except that the cell differentiation rate δ_2 is defined as a function of viral load V. Note that defining the cell death rate κ_2 instead of δ_2 as a function of V may produce similar results. For "HIV^{-}", parameters are set as follows on the basis of obtained data shown in Figs. 1-10. δ1=0.7, δ2=1.5, δ3=3.5, σ1=0.0000002, σ2=0.000002, σ3=0.0002, σ4=0.0002, ρ1=0.035, ρ2=0.046, ρ3=0.003, ρ4=0.001, κ1=0, κ2=0, κ3=0, κ4=0.1. For "HIV^{+}", the following parameters are further set. ν1=0.01, ν2=-0.01, ν3=0.05, ν4=0.11, r=1.3, d=0.3, k=0.5. In the panel "HIV^{+} δ(V)/κ(V)", the following parameter is redefined. δ2=1.5−0.0001V. Redefinition of κ(V) is omitted as this produces similar results.
